# Mechanistic Insights and Technical Challenges in Sulfur-Based Batteries: A Comprehensive *In Situ*/*Operando* Monitoring Toolbox

**DOI:** 10.1021/acsenergylett.4c02703

**Published:** 2024-12-04

**Authors:** Jing Yu, Ivan Pinto-Huguet, Chao Yue Zhang, Yingtang Zhou, Yaolin Xu, Alen Vizintin, Juan-Jesús Velasco-Vélez, Xueqiang Qi, Xiaobo Pan, Gozde Oney, Annabel Olgo, Katharina Märker, Leonardo M. Da Silva, Yufeng Luo, Yan Lu, Chen Huang, Eneli Härk, Joe Fleming, Pascale Chenevier, Andreu Cabot, Yunfei Bai, Marc Botifoll, Ashley P. Black, Qi An, Tazdin Amietszajew, Jordi Arbiol

**Affiliations:** † Catalan Institute of Nanoscience and Nanotechnology (ICN2), CSIC and BIST, Barcelona 08193, Spain; ‡ Catalonia Institute for Energy Research (IREC), Barcelona 08930, Spain; § School of Physical Science & Technology, Lanzhou University, Lanzhou 730000, China; ∥ Zhejiang Key Laboratory of Petrochemical Environmental Pollution Control, National Engineering Research Center for Marine Aquaculture, Marine Science and Technology College, Zhejiang Ocean University, Zhoushan, Zhejiang Province 316004, China; ⊥ Department of Applied Physics, Aalto University, Espoo 00076, Finland; # National Institute of Chemistry, Ljubljana 1000, Slovenia; ∇ ALBA Synchrotron, Barcelona 08290, Spain; ○ College of Chemistry and Chemical Engineering, Chongqing University of Technology, Chongqing 400054, China; ⧫ State Key Laboratory of Applied Organic Chemistry, Lanzhou University, Lanzhou 730000, China; ¶ Univ. Grenoble Alpes, CEA, CNRS, Grenoble INP, IRIG, SYMMES, Grenoble 38000, France; †† Univ. Grenoble Alpes, CEA, IRIG, MEM, Grenoble 38000, France; ‡‡ Department of Chemistry, Federal University of Jequitinhonha e Mucuri, Diamantina 39100-000, Brazil; §§ Department of Applied Biology and Chemical Technology, Hong Kong Polytechnic University, Hong Kong, China; ∥∥ Institute of Electrochemical Energy Storage, Helmholtz-Zentrum Berlin für Materialien und Energie, Berlin 14109, Germany; ⊥⊥ Department of Chemistry, University of Barcelona, Barcelona 08028, Spain; ## Centre for E-Mobility and Clean Growth, Coventry University, Coventry CV1 5FB, United Kingdom; ∇∇ Institut de Ciència de Materials de Barcelona (ICMAB-CSIC), Barcelona 08193, Spain; ○○ School of Materials and Energy, Yunnan University, Kunming 650091, China; ⧫⧫ ICREA, Pg. Lluis Company, 08010 Barcelona, Spain

## Abstract

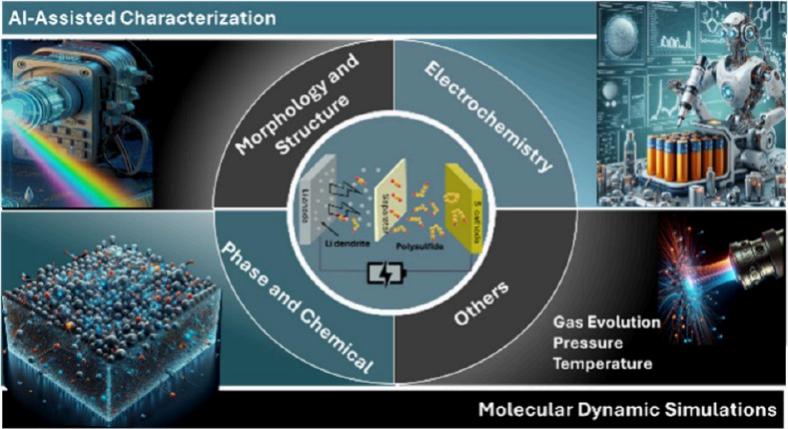

Batteries based on sulfur cathodes offer a promising energy storage solution due to their potential for high performance, cost-effectiveness, and sustainability. However, commercial viability is challenged by issues such as polysulfide migration, volume changes, uneven phase nucleation, limited ion transport, and sluggish sulfur redox kinetics. Addressing these challenges requires insights into the structural, morphological, and chemical evolution of phases, the associated volume changes and internal stresses, and ion and polysulfide diffusion within the battery. Such insights can only be obtained through real-time reaction monitoring within the battery’s operational environment, supported by molecular dynamics simulations and advanced artificial intelligence-driven data analysis. This review provides an overview of *in situ/operando* techniques for real-time tracking of these processes in sulfur-based batteries and explores the integration of simulations with experimental data to provide a holistic understanding of the critical challenges, enabling advancements in their development and commercial adoption.

Sulfur cathodes are at the cutting edge of energy storage technology, offering a solution for the development of batteries with much higher energy densities compared to conventional lithium-ion batteries. In particular, lithium–sulfur batteries (LSBs), with a theoretical energy density of approximately 2600 Wh kg^–1^, nearly five times that of lithium-ion batteries, leverage the high capacity of sulfur (1675 mAh g^–1^) and the low atomic weight of lithium.^1^ Moreover, sulfur is abundant, inexpensive, nontoxic, and safe, making sulfur cathodes an attractive candidate for both mobility and stationary energy storage applications. Despite these advantages, the commercialization of batteries based on sulfur cathodes, referred to here as sulfur-based batteries, excluding those using sulfide electrolytes with other cathodes, is hindered by substantial technical challenges at the cathode, including the following:

(1) Polysulfide dissolution and shuttle effect. Within LSBs based on conventional ether-based liquid electrolytes, during the discharge process, sulfur is converted into soluble lithium polysulfides (LiPSs, Li_2_S_*x*_, 4 ≤ *x* ≤ 8) that dissolve in the electrolyte, resulting in the loss of active cathode material and reduced ionic conductivity.^2^ This leads to capacity fading, shortened cycle life, and lower charge/discharge rates. These dissolved polysulfides can migrate to the lithium anode, where they are reduced to insoluble Li_2_S_2_/Li_2_S. During charging, these species are reoxidized into soluble polysulfides that return to the cathode, creating a continuous shuttling effect. This shuttling is akin to an internal short circuit, contributing to self-discharge and reduced Coulombic efficiency. The ongoing cycle of dissolution and precipitation ultimately causes active material loss and the formation of insulating layers on the electrodes, further degrading battery performance.
(2) Low electrical conductivity of sulfur (5 × 10^–30^ S/cm) and its discharge products, e.g., Li_2_S and Li_2_S_2_ (3.4 × 10^–7^ S/cm). The insulating sulfur and metal sulfides (M_*x*_S) formed during charging and discharging passivate the cathode surface, hindering the complete reaction of the active material, and thereby limiting the achievable capacity. This effect is further exacerbated by the highly inhomogeneous nucleation and growth of S_8_ and metal sulfides, leading to the formation of large precipitates that are never fully reoxidized/reduced, resulting in additional capacity loss.^3^
(3) Substantial volume changes of sulfur during oxidation and reduction cycles, ca. 80% in the most favorable LSB case, associated with the incorporation of the metal and amplified in the case of Li and Na by density differences between sulfur (2.03 g/cm^3^) and the metal sulfides (e.g., Li_2_S at 1.67 g/cm^3^).^4^ When sulfur is fully reduced to metal sulfide, it undergoes a substantial volume expansion that induces mechanical stresses and structural degradation of the electrode, further compromising the battery’s stability and performance. At the same time, during the sulfur oxidation, the decrease in volume may disconnect part of the active material from the electronic transport framework. This detachment prevents those regions from participating further in the reaction, effectively reducing the overall capacity of the battery.

Figure 1 illustrates the electrochemical processes. Table 1 outlines the key sulfur-related parameters that need to be understood to optimize cell components and enhance the overall battery performance. The primary challenge for conversion-type cathodes like sulfur lies in the dynamic reorganization of the active material during each charge and discharge cycle. Consistent participation of all the active material in each cycle requires highly uniform nucleation and growth of the different phases throughout cycling. Critical parameters to be understood and adjusted include nucleation sites, nucleation and growth dynamics, and the role of catalysts. Time-resolved analyses with 3D spatial resolution are required to determine the evolution of the unknowns within the porous/nanocomposite 3D nature of the cathode material. Additionally, monitoring the evolution of the electrolyte composition during cycling, specifically in terms of polysulfide presence, lithium-ion concentration, and potential degradation products, is crucial. The electrolyte’s wetting properties on the cathode and the distribution of lithium ions within the cathode material are also essential factors to assess and optimize. Monitoring the presence of polysulfides in the electrolyte and their possible interaction with the anode surface is also critical.

**Figure 1:**
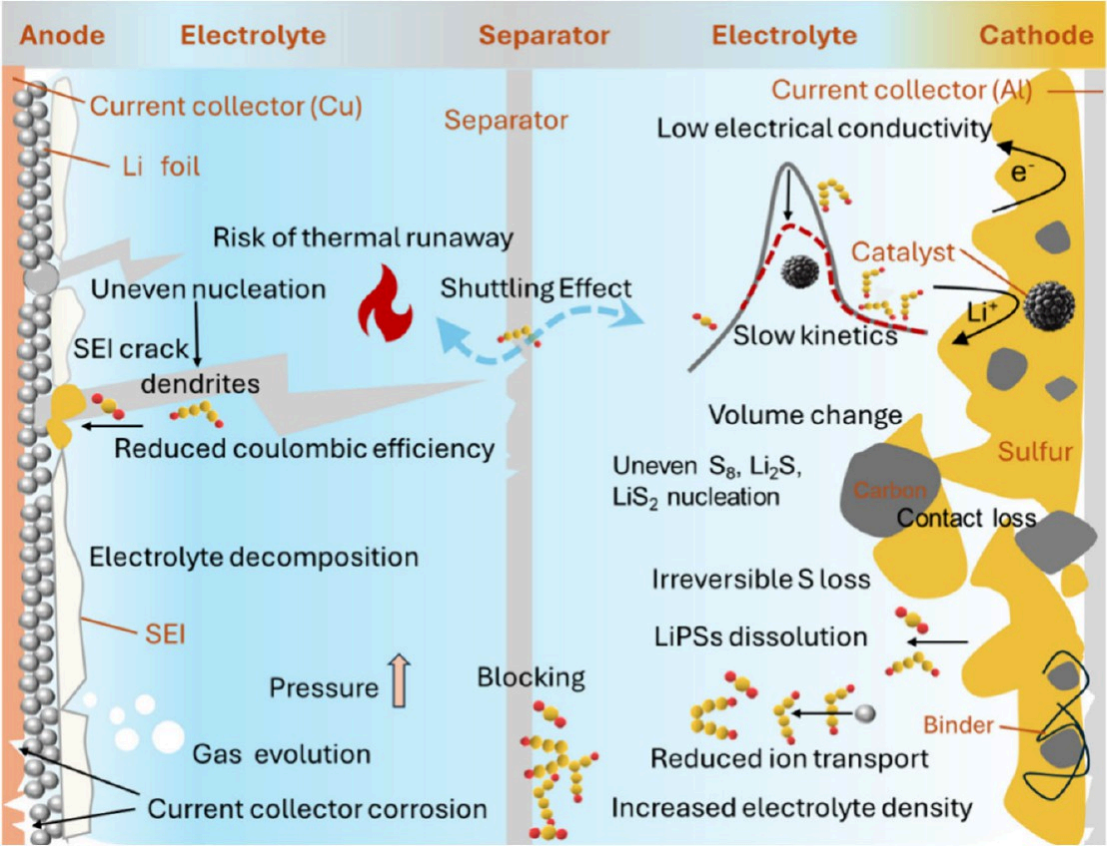
Illustration of the intricate electrochemical processes, degradation mechanisms, and structural and chemical transformations occurring in sulfur-based batteries, exemplified by the LSB.

**Table 1:** Key Unknowns in Sulfur-Based Batteries, Required Observations, and Related Time-Resolved *In Situ*/*Operando* Techniques^a^

	**Unknown**	**Observable**	**Technique**
**Cathode**	3D dynamic structural evolution of cathode composite	Sulfur and metal sulfide consumption/nucleation/growth, compositional gradient, domain size, and shape, nucleation sites, carbon reorganization, pore filling, and volume changes. Diffusion depth of metal ions within the cathode material and the active sulfur.	XTM, TXM, XAS, SEM, NI, SAXS, SANS, OM, AFM, MD
	Intermediates in the S_8_ + MΔM_*x*_S reaction.	Identification of species, chemical states, and concentration at the cathode, interphase, and electrolyte. 3D spatial distribution and local gradients. Diffusion of metal polysulfides within the cathode.	Raman spectroscopy, EIS, XRD, XPS, NI, NMR, EPR, FTIR, UV–vis
	Catalyst role and evolution	Catalyst phase, composition, surface chemistry, electronic structure, and morphology.	XPS, XAS, (S)TEM, MD
	Reaction mechanism	Identification of chemical species, adsorption/reaction sites and coverage, electronic interaction, bond formation, charge redistribution/transfer, limiting reaction steps, M-S reaction catalytic mechanism. Parasitic reactions.	XPS, XAS, (S)TEM, MD, gas, temperature, and pressure sensing
	Ionic and electronic charge transport	Electrical conductivity of the different phases, electrical interconnectivity within cathode composite. Charge transfer process. Charge, potential, resistivity, and metal-ion diffusivity mapping.	EIS, AFM, NMR, (S)TEM, XAS
**Electrolyte**	Chemical and electrochemical stability	Identify and quantify degradation products in the electrolyte, concentration of metal ions, gas generation, and accumulation.	NMR, FTIR, EPR, UV–vis, MD, gas and pressure sensing
	Ionic transport	Electrolyte spatial distribution, ionic conductivity, ion gradients, contact electrolyte/electrode, wettability of electrode, and ionic transport mechanism.	NMR, EIS, NI, EPR
**Anode**	Degradation	Sulfur presence at the anode surface	FTIR, XPS
**Other components and overall cell**	Separator	Degradation products. Separator structural properties and composition, pore filling, and ionic conductivity.	TXM, NI, FTIR, NMR, SEM, (S)TEM
	Current collector stability	Current collector surface morphology, composition, and degradation products.	NI, XTM, NMR, SEM, XPS
	Cell state of health	Swelling, temperature, pressure, stress, current and voltage distribution within the cell.	OM, temperature, gas, and pressure sensing

a Abbreviations: X-ray tomography (XTM); transmission X-ray microscopy (TXM); (scanning) transmission electron microscopy ((S)TEM); scanning electron microscopy (SEM); atomic force microscopy (AFM); optical microscopy (OM); neutron imaging (NI); X-ray diffraction (XRD); small-angle neutron scattering (SANS); small-angle X-ray scattering (SAXS); X-ray photoelectron spectroscopy (XPS); ultraviolet–visible (UV–vis) spectroscopy; nuclear magnetic resonance (NMR); Fourier-transform infrared spectroscopy (FTIR); X-ray absorption spectroscopy (XAS); electron paramagnetic resonance (EPR); molecular dynamics (MD) simulations.

*Ex situ* and *post-mortem* characterization methods, which analyze battery components outside their operational environment, offer detailed useful snapshots of battery components but fall short in capturing real-time dynamics and material/component interaction in real operation conditions. This limitation reduces not only the comprehensiveness but also potentially the accuracy of the results as side reactions during sample preparation can distort the structure-to-performance relationships, potentially leading to misleading conclusions. In this direction, sample handling and specific instrument conditions may compromise sample integrity and introduce artifacts. *Ex situ* and *post-mortem* analyses require treating the sample to adapt it to the characterization technique. This preparation often involves exposure to atmospheres different from the battery’s internal environment and processing steps such as washing or drying within an inert gas environment to remove residual surface electrolytes that could interfere with the analysis. Moreover, the operational environment of the characterization, often involving vacuum or specific conditions, can for instance alter or remove surface polysulfides through dissolution, further contributing to artifacts in the results.

To overcome these limitations, *in situ*, *quasi-operando* and *operando* techniques have emerged as powerful tools for real-time probing of dynamic processes. These techniques enable direct observation of electrochemical reactions and structural changes during battery operation with all interacting components, providing critical insights into the mechanisms driving the performance and degradation of sulfur-based batteries, insights that are essential for the rational development of next-generation battery technologies.^5^

*Operando* specifically refers to real-time analysis of battery components during active operation, such as during charging and discharging cycles, providing direct observation of system behavior under actual working conditions. In contrast, *in situ* is a broader term that involves the analysis of components within their original operational environment, even when the system is not actively operating, allowing for pauses in the process of acquiring data.^6^

Several *in situ* and *operando* characterization techniques have been employed to study the components and processes of sulfur-based batteries. The morphological evolution of sulfur, lithium sulfides, solid electrolyte interphase (SEI) layers, and lithium during cycling has been tracked using (S)TEM, SEM, AFM, OM, XTM, and NI. Reaction mechanisms and the kinetics of sulfur conversion to LiPSs and subsequently to Li_2_S have been examined using *in situ*/*operando* techniques such as XAS. Additionally, *in situ* XPS and electron energy loss spectroscopy (EELS) provide insights into the chemical environment of various elements within the cell during charge and discharge processes. To monitor rapid changes in polysulfides during battery operation, UV–vis, Raman spectroscopy, NMR, FT-IR, and EPR yield highly valuable information. Each of these *in situ*/*operando* techniques has specific analytical requirements, along with distinct strengths and limitations. Therefore, selecting the most appropriate technique or a combination of techniques according to the research objectives is crucial for advancing the study of sulfur-based batteries, elucidating underlying mechanisms, and addressing technical challenges.

This review covers the principles of *in situ* and *operando* characterization techniques, the specific setups used for testing sulfur-based batteries, and relevant case studies where these techniques have been applied. The integration of *in situ* and *operando* characterization techniques with advanced computational modeling offers significant potential to deepen our understanding of sulfur-based batteries. DFT and DM simulations can complement experimental observations by providing atomic-level insights into the energetics and kinetics of electrochemical reactions. By correlating *in situ* findings with computational models, researchers can develop more precise representations of battery processes, leading to more rational material design and optimization strategies. Furthermore, machine learning algorithms can efficiently analyze large data sets from *in situ* and *operando* experiments, uncovering patterns and correlations that might be overlooked with traditional analysis methods, thereby accelerating the discovery of critical insights.

## Morphological and Structural Characterization

Monitoring and understanding the numerous structural changes occurring within a battery during the first few discharge/charge cycles, such as the reconstruction of cathode material catalysts, M_*x*_S/S deposition from the S/M_*x*_S conversion, volume expansion, and crack development, are critical for optimizing battery performance and safety. The continuous chemical and structural transformations occurring at the electrode–electrolyte interface demand advanced methods capable of real-time morphology monitoring. These morphological changes provide intuitive insights into intermediate products, which are essential for understanding the underlying mechanisms of sulfur-based batteries, particularly in the sulfur cathode. A range of advanced *in situ*/*operando* scanning, mapping, and imaging techniques have been particularly applied to LSB research, including SEM, (S)TEM, AFM, OM, XTM, and NI, among others. This chapter provides an overview of each technique and highlights the key aspects of battery behavior that can be monitored using these methods.

### Scanning Electron Microscopy (SEM)

SEM generates images of a sample using a focused electron beam, where the image is formed by correlating beam position with signal intensity. The interaction of electrons with the material produces various signals, which can be analyzed to provide valuable information on the sample’s composition, morphology, and topography. A main challenge in *in situ* SEM analysis for sulfur-based batteries lies in the development of the cell adapted to the electrolyte serving as the ion transfer medium, which may be in liquid, gel, or solid form. Additionally, alkali metals’s highly reactive nature requires the use of complex systems that exclude water, oxygen, and nitrogen to transfer the battery into the electron microscope. These systems must also be compatible with vacuum or noble gas environments to ensure safe and effective handling.

Conducting *in situ* SEM investigations of sulfur-based batteries in vacuum requires the use of an electrolyte that is compatible with this environment. Under vacuum conditions, sulfur sublimation can lead to its redistribution, resulting in measurement artifacts, as well as permanent vacuum chamber contamination,^7^ hindering the overall system understanding. To prevent sulfur sublimation during *in situ* SEM characterization, specialized techniques are necessary. Glass-ceramic^8^ or polymeric solid electrolytes^9^ allow observation of LSBs during operation by SEM, providing valuable insights into phenomena such as polysulfide dissolution, the formation of sulfur-rich insulating layers on the lithium anode, and the appearance of S_8_ during charge/discharge cycles. When combined with UV–vis spectroscopy, these studies revealed the accumulation of various polysulfides, including Li_2_S, during cycling.^10^ Specifically, S_6_ polysulfides were formed during charging, while S_4_ polysulfides appeared during discharging, accompanied by a polysulfide shuttle mechanism. Although polymer electrolytes do not completely prevent lithium loss from the anode, they reduce catholyte dissolution and mitigate polysulfide displacement, contributing to improved system stability.

The use of liquid electrolytes in SEM is more challenging due to vacuum compatibility, requiring the use of low vapor pressure electrolytes in an open-cell configuration. Ionic liquid-based electrolytes offer a solution, as they allow for the investigation of electrochemical systems under vacuum conditions and are compatible with standard SEM instruments.^11^ However, they come with certain drawbacks, such as lithium plating in the non-electron-conductive electrolyte and Coulomb interactions that can lead to local electrode flooding. Despite these limitations, this approach provides a valuable opportunity to study electrodes under working conditions using SEM-based methods.

**Figure 2:**
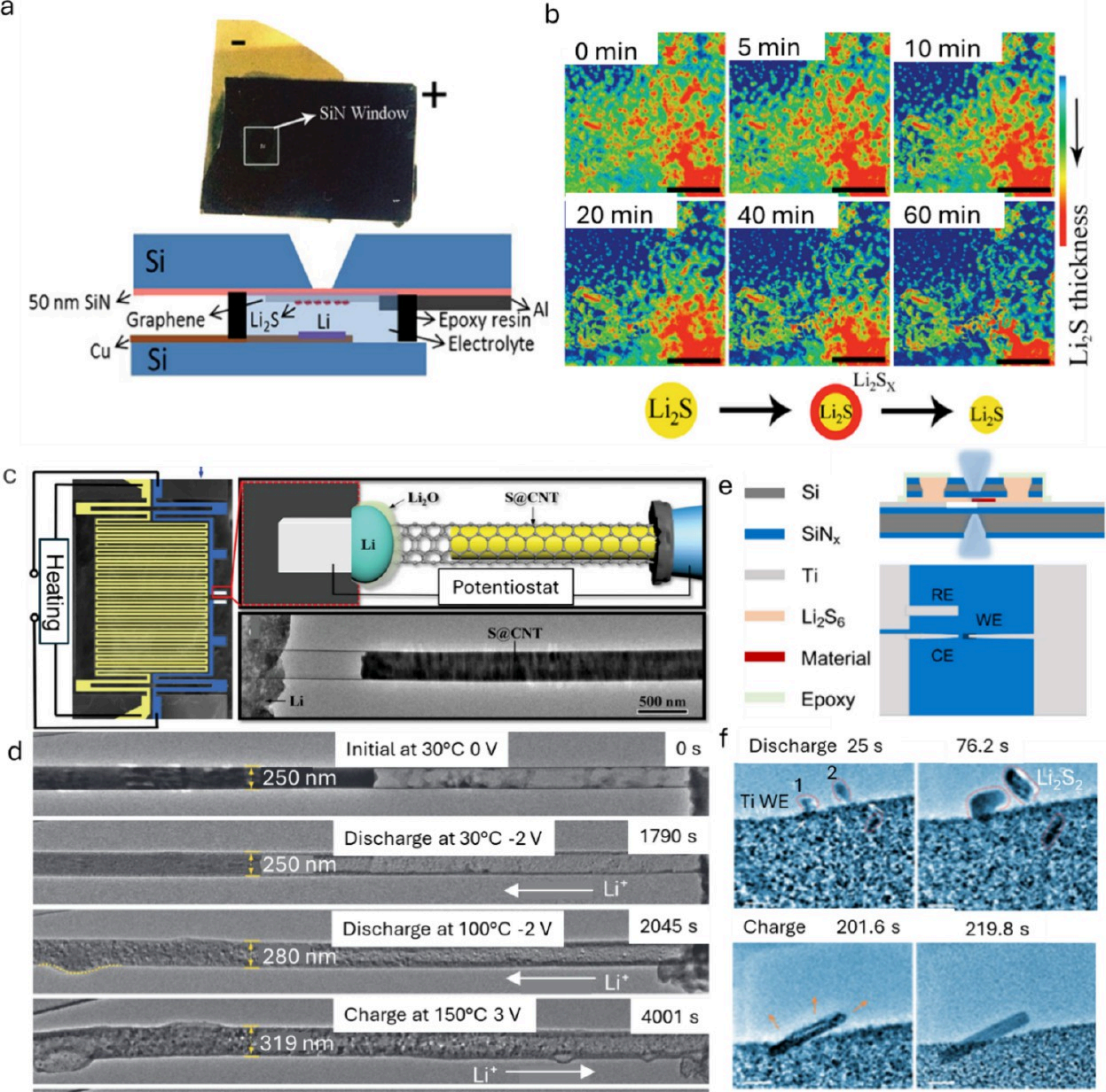
(a) Schematic of an electrochemical microcell for *in situ* SEM analysis. (b) Time-lapse SEM images of the activation process of Li_2_S on a single-layered graphene electrode in a standard lithium bis(trifluoromethanesulfonyl)imide (LiTFSI) 1,2-dimethoxyethane (DME)/1,3-dioxolane (DOL) electrolyte. Scale bar = 20 mm. Reproduced with permission from ref (12). Copyright 2015, Wiley-VCH. (c) Scheme showing the setup of the solid cell implemented with a microelectromechanical system heating device for *in situ* TEM observation. (d) TEM images showing lithiation of S@CNT at different temperatures. Reproduced with permission from ref (14). Copyright 2020, Wiley-VCH. (e) Schematic illustration of an electrochemical *in situ* liquid TEM cell from the top and side views, composed of top/bottom chips with 10 nm SiN_*x*_ observation windows, 100 nm spacers, (counter/reference electrodes: Ti). (f) Time-series TEM images of Li_2_S deposition (dashed red frame) and dissolution (dashed blue frame) in an electrochemical liquid cell. Scale bars = 200 nm. Reproduced with permission from ref (1). Copyright 2023, Springer Nature.

Alternatively, the use of electron-transparent silicon-nitride (SiN_*x*_) windows (Figure 2a) to separate the electrolyte from the vacuum environment, allows the use of high vapor-pressure liquids, such as most organic electrolytes commonly found on LSBs.^12^ The setup consists of a top silicon chip frame with a SiN_*x*_ membrane, just tens of nanometers thick, serving as the viewing window. In this closed-cell configuration, the cell is filled with liquid electrolyte and sealed. A variation of this strategy involves placing the SiN_*x*_ window at the end of the microscope column, enabling controlled atmosphere operation in the full microscope chamber, such as in ambient pressure SEM.^13^ This approach enables the study of sulfur-based batteries under conditions closer to real-world battery systems, providing relevant insights into electrode behavior during operation.

*In situ* SEM enables the observation of dynamic changes in LiPSs as they gradually dissolve into the electrolyte, as well as the size variations of Li_2_S particles during the electrochemical process.^12^ It also allows for the observation of morphological changes, such as porosity at the electrode/electrolyte interface, from the cross-section view.^10^

### (Scanning) Transmission Electron Microscopy ((S)TEM)

(S)TEM offers more detailed insights into the morphology, structure, and even chemical composition of samples and interfaces at higher resolution, enabling both direct imaging and spectroscopic analysis.

For effective and reliable microscopy imaging, it is essential to ensure the sample’s integrity during monitoring, ensuring it remains unaffected by the high vacuum, intense electron beam, high electric field, and other extreme conditions within the electron microscope. *Operando* TEM characterization of sulfur-based batteries is particularly challenging due to several factors: the sublimation of sulfur, the volatility of the electrolyte, and the susceptibility of liquid-phase polysulfides to electron-induced damage. Additionally, the need for samples to be thin enough for electron transmission poses a significant technical challenge that must be addressed to achieve accurate and meaningful results.

Existing *in situ* holder setups previously used for LSB research often feature sulfur deposited on the grid as the cathode or encapsulated in carbon nanotubes, and a small amount of lithium metal on a probe (tungsten/copper) as the anode. In most previous work, a thin layer of oxidized Li, Li_2_O, on the Li surface, serves as a solid electrolyte, transferring Li^+^. This setup has been used to observe the formation progress of Li_2_S precipitation by temperature (Figure 2c,d),^14^ the lithiation-induced expansion accommodated by the remaining pore volume,^15^ and volume expansion.^4^ Such observations mainly highlight the relationship between cathode volume expansion and electrochemical performance.^16^ However, TEM setups relying on partially oxidized Li metal forming a Li_2_O solid electrolyte are unable to capture sulfur losses by dissolution in liquid electrolyte. Levin et al. addressed sulfur sublimation by using *in situ* cryo-TEM at −173 °C, which ensured sulfur stability and demonstrated cryo-TEM as an excellent technique for cathode characterization in LSBs.^7^ However, liquid electrolytes could not be studied in this setup either.

Zhou et al. achieved a significant breakthrough by constructing a Li–S liquid nanocell and using TEM to observe real-time LiPS evolution on the electrode surface at the atomic scale (Figure 2e). They discovered two distinct modes of Li_2_S deposition and dissolution during potentiostatic discharge: single-step rod- or plate-shaped Li_2_S deposition, and two-step deposition involving metastable Li_2_S_2_, as illustrated in Figure 2g. Interestingly, when Mo nanoclusters/N-doped graphene were introduced, rod-shaped Li_2_S nucleation was suppressed. In the two-step deposition process, solid nanocores form at the electrode–electrolyte interface during the initial nucleation phase, acting as preferential seeds for further grain growth (as seen in particles 1 and 2).^1^ This study represents a pioneering breakthrough in the direct observation of sulfur-based batteries at the atomic scale, enabling a detailed understanding of how catalysts influence the nucleation process.

It is worth noting that electron beam damage, including atomic displacement, electrostatic charging, and movement or reactions in particles or the liquid electrolyte during morphology monitoring, can be mitigated to some extent by using low-energy beams or low-dose measurements. However, since beam energy and dose are directly tied to resolution, finding the optimal balance is critical to achieving reliable results without sacrificing detail.

### Atomic Force Microscopy (AFM)

AFM uses a sharp tip on a cantilever to scan the surface of a sample, measuring the forces between the tip and the sample to generate detailed images with nanometer resolution. *In situ* AFM is often combined with OM or Raman spectroscopy to provide a more comprehensive understanding of interfacial issues in sulfur-based batteries. This combination is particularly valuable for monitoring the morphological and chemical evolution of the battery components.

**Figure 3:**
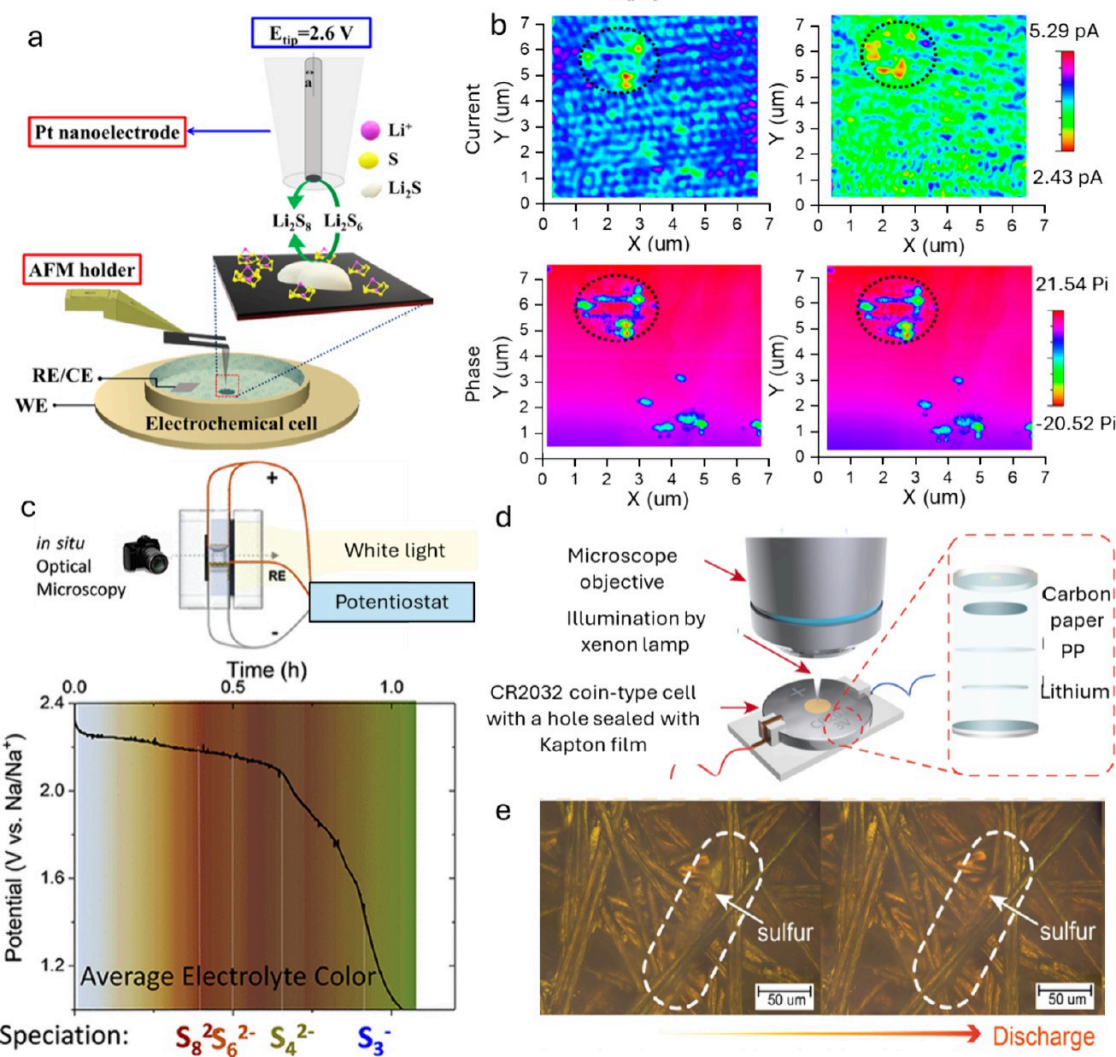
(a) Schematic representation of an AFM-scanning electrochemical microscopy electrochemical cell setup. The zoom-in part depicts the SECM mode used for imaging the cathode surface. (b) AFM-scanning electrochemical microscopy electrochemical imaging of Li_2_S/Li_2_S_2_ on carbon surface during oxidation: current (first row), and phase shift (second row) mapping of Li_2_S/Li_2_S_2_ surface. The first column images correspond to Li_2_S/Li_2_S_2_ galvanostatically deposited on glassy carbon before oxidation. The second column images correspond to the Li_2_S oxidation at substrate potentials of 2.7 V vs Li/Li^+^ respectively. *E*_tip_ = 2.6 V. Reproduced with permission from ref (17). Copyright 2019, American Chemical Society. (c) Optical electrochemical *in situ* cell for sodium sulfur batteries (up); optical signal and its corresponding discharge profile (down). Reproduced with permission from ref (22). Copyright 2020, American Chemical Society. (d) Coin cell type. (e) *In situ* OM images showing the dissolution and formation of sulfur crystals during the discharging process. Reproduced with permission from ref (29). Copyright 2022, Elsevier.

AFM-scanning electrochemical microscopy enables simultaneous monitoring and correlation of the morphology of interfacial M_*x*_S with the electrochemical oxidation behavior. As an example, Mahankali et al. designed a four-electrode electrochemical cell where the AFM-scanning electrochemical microscopy tip served as the working electrode 1, a glassy carbon circular disk (∼4 mm^2^ area) was used as the working electrode 2, and a lithium strip acted as both reference and counter electrode, with an electrolyte of LiTFSI and LiNO_3_ (Figure 3a).^17^ They distinguished conducting and insulating regions on Li_2_S particles during oxidation using *in situ* electrochemical and alternating current phase mappings. During charging, the conductive part dissolves, while the insulating part reacts with intermediate LiPSs. At higher oxidation potentials, the reacted LiPSs turn into insulating products, which accumulate during cycling, resulting in reduced utilization of active materials and ultimately capacity decay (Figure 3b).

*In situ* AFM can also quantitatively assess the SEI properties of sulfur-based batteries, providing detailed information on 3D nanoscale morphology, film thickness, mechanical modulus, and local ionic conductivity. As an example, Li et al. introduced 1,3,5-trioxane as a reactive cosolvent that decomposes on the lithium anode surface, incorporating organic components into the SEI. This process effectively suppresses parasitic reactions with lithium LiPSs and protects the lithium metal anode, enabling long-cycle performance for LSBs.^18^ Similarly, Hou et al. utilized 1,3,5-trioxane (TO) and DME as electrolyte cosolvents to develop a highly mechanically stable SEI in LSBs. By using *in situ* AFM combined with other techniques, the authors demonstrated that TO, with its high polymerization capability, decomposes to form an organic-rich SEI, improving mechanical stability and thereby mitigating cracking and regeneration. As a result, the consumption rates of active lithium, lithium polysulfide, and electrolyte are reduced, enhancing overall LSB performance.^19^

Liu et al. used *in situ* AFM combined with OM to reveal that SEI formation in an electrolyte with added LiNO_3_ occurs in two stages. Initially, loose nanoparticles (≈102 nm) form at the open circuit potential, followed by the generation of dense nanoparticles (≈74 nm) during discharge, driven by the combined effect of LiPSs and LiNO_3_. This dense SEI film not only prevents LiPS corrosion but also promotes uniform lithium deposition, thereby enhancing the electrochemical performance of quasi-solid-state (SS) LSBs.^20^

Evidence from *in situ* AFM provides valuable insights into how electrolyte additives affect SEI composition, offering crucial information for interface engineering and electrolyte design in LSBs. Additionally, there have been significant breakthroughs in applying *in situ* AFM for cell characterization when combined with other techniques, such as in the environmental transmission electron microscopy–AFM platform, which holds great potential for application in LSBs and could be realized in future studies.^21^

### Optical Microscopy (OM)

*In situ* OM is an effective tool for observing the dynamic aging process of sulfur-based batteries at various scales, from centimeters down to microns. This technique relies on sulfur and metal polysulfides being colorful species. Usually, a darker color corresponds to a higher-order polysulfide, except for the colorless S_8_ and S^2–^, as illustrated by Carter et al. studying sodium–sulfur batteries using an H-type cell (Figure 3c).^22^ OM offers several advantages, including a flexible operating environment, a simple experimental setup, and high reliability. These features have made it the most widely used *in situ* characterization method among researchers. A variety of cell architectures has been used for *in situ* OM analysis, including H-cells, glass plate clamps,^23^ glass colorimetric dishes,^24^ glass tubes,^25^ capillaries,^26^ button cells,^27^ and pouch cells.^28^ Selecting the appropriate reaction cell is crucial and should be guided by the specific requirements of the test and the targeted information to be obtained.

Zheng employed *operando* OM assembled from CR2032 coin-type cells to systematically study the evolution of solid sulfur species on carbon fiber within an LSB.^29^Figure 3d depicts their *operando* observation platform for coin type. At the start of discharge, amber-like sulfur crystals appeared on the carbon fiber (Figure 3e). As discharge continued, the sulfur crystals dissolved, eventually vanishing, and the electrolyte gradually darkened, indicating the solid–liquid conversion from S_8_ to S_8_^2–^ in the LSB cell. During charging, bulk sulfur crystals formed within the electrode, with the electrolyte color darkening due to higher-order LiPS generation.^30^

By correlating color or contrast changes with polysulfide spices, the distribution of polysulfide in the electrolyte can be estimated *in situ* in a nondestructive, rapid, and cost-effective way. This method is highly accessible, being intuitive, fast, simple, and affordable. However, it is limited to detecting low polysulfide concentrations, as the separator quickly reaches color saturation. Additionally, a completely transparent, colorless electrolyte is necessary to ensure that any observed color changes are solely due to polysulfide diffusion. The color changes are often relative, with specific polysulfide species displaying distinct color codes, such as S_6_^2–^ is orange and S_3_^•–^ is blue, though these are strongly influenced by concentration. Therefore, the intricate interconversion of polysulfide species, coupled with the complex electrolyte composition, gradient colors, and concentration effects, makes it difficult to differentiate individual species accurately.

OM also falls short in operating within visible light wavelengths, offering limited imaging resolution and tracking capabilities, which constrains the detection of species such as Li_2_S. Therefore, future improvements and innovations are essential. For example, by applying optical fluorescence microscopy taking advantage of the spatially resolved fluorescence intensity correlated with a quantitative assessment of the polysulfide concentration in the electrolyte. Additionally, combining *in situ* OM with Raman spectroscopy provides more comprehensive insights into the evolution of material crystal structure, elemental composition, and morphology during battery operation.

### Transmission X-ray Microscopy (TXM), X-ray Radiography, and X-ray Tomography (XTM)

TXM is a nondestructive technique for analyzing morphological changes with a high spatial resolution, which has also been applied to sulfur-based batteries.^31^ Unlike electron microscopy, TXM does not require particular sample preparation and allows for the penetration of thicker sample layers while maintaining high spatial resolution. The typical sample sizes range from ∼50 μm to the entire cell stack, with spatial resolution from ∼20 nm to 10–30 μm for large batteries. Additionally, TXM offers a broad field of view.^32^ TXM operates on a synchrotron beamline using a Fresnel lens to focus the X-ray beam on a very small zone, determining the resolution. The transmitted beam is recorded on a detector while scanning the zone of interest, producing an image. Since sulfur and carbon have similar X-ray absorption properties, the beam energy has to be tuned to maximize contrast (5–8 keV).^33^ Alternatively, using a ring detector and phase contrast imaging can yield good results. With its short acquisition time, TXM is particularly well-suited for *operando* studies.

The initial application of *operando* TXM in sulfur-based battery research was marked by Nelson et al.,^33^ who studied an LSB cycled at C/8 in a pouch cell configuration. This study achieved a spatial resolution of 30 nm and a field of view of 15–30 μm. They tracked individual particles and calculated polysulfide loss to the electrolyte via X-ray absorption contrast. Their findings revealed that during the first discharge, particles slightly expanded and, at the same time, lost active material due to polysulfide dissolution, forming smaller, more porous particles. Subsequent charging did not increase particle size or form new particles, suggesting that most active material remains intact. However, based on the capacity fade, they conclude that even minimal polysulfide dissolution into the electrolyte significantly affects electrochemistry.

Contrary to this, Lin et al.^34^ presented a more intricate picture of polysulfide dissolution and redeposition in LSB cells using *operando* TXM. Their detailed observations demonstrated that particle expansion and shrinkage rates varied depending on the operating protocol and lithiation stage. Eventually, particles ranging from 3 to 12 μm disappeared and were replaced by large insoluble polysulfide crystals (Li_2_S_2_, Li_2_S). Their *operando* observations revealed that existing particles grew before new ones formed, indicating that larger undissolved particles would grow with subsequent cycling, reducing the number of active particles and increasing average particle size.

Another nondestructive X-ray-based imaging technique is X-ray radiography, which uses X-ray absorption contrast at a large scale on a whole cell. This technique, available from laboratory X-ray sources or on synchrotron beamline, efficiently probes cathode materials at the electrode scale.

Risse et al.^35^ performed *operando* analysis of a sulfur cathode using this method in a coin-type operating cell combined with EIS. Following this, Jafta et al.^36^ used *operando* X-ray radiography to explore the relationship between the macrostructure of the carbon matrix and the electrochemical performance of sulfur cathodes. Their findings demonstrated that an open structure with larger voids more effectively accommodates the volume changes of sulfur, thereby enhancing overall electrochemical performance. The radiographic analysis by Jafta et al.^36^ revealed a “breathing” mechanism characterized by the expansion and contraction of LiPSs. LiPSs are pushed to the electrode’s edges during discharge, while they migrate back toward the center during charge. This cyclic movement was found to cause an inactive sulfur region at the edges over time and with increased cycling rates, contributing to capacity fading.

Contrary to the cited techniques, XTM enables the analysis of a sample’s complete 3D volume by combining a series of 2D radiographs, uncovering microstructural changes along a third axis that are invisible to 2D techniques.^37^ In this method, the sample is placed between the X-ray source and a flat panel detector as in radiography. Hundreds of images are captured as the sample rotates along an axis perpendicular to the beam, and these images are reconstructed into a 3D image using specialized software. XTM can be performed on laboratory computed tomography apparatus or synchrotron beamline, depending on the targeted resolution and sample size, ranging from full cells^38^ to resolutions as fine as a few tens of nanometers.^39^ Achieving the highest resolution, however, typically requires a small *ex situ* sample or a miniaturized cell specifically designed for *operando* analysis.^39,40^

In X-ray absorption computed tomography, contrast is obtained through absorption, enhancing the visualization of different densities and compositions. XRD computed tomography, on the other hand, uses the diffraction contribution to the X-ray attenuation coefficient each time a grain fulfills the diffraction condition, providing detailed atomic structure information and yielding high contrasts among materials with similar absorption coefficients.^41^

Yermukhambetova et al.^42^ were the first to conduct 3D *in situ* tomography on Li–S cells using a lab micro- computed tomography instrument. With a pixel size of 780 nm and a field of view of 750 μm, they identified an uneven distribution of the sulfur phase fraction along the electrode thickness. After 10 cycles, sulfur agglomeration and depletion near the separator were evident. Further highlighting sulfur distribution heterogeneity, Tan et al.^43^ used X-ray phase contrast micro- computed tomography to quantify the solid sulfur phase in the cathode. Their study demonstrated the preferential formation of sulfur clusters during cycling, starting from an initially well-dispersed cathode.

The capabilities of *in situ*/*operando* tomography are significantly enhanced when combined with other characterization tools, providing complementary insights. Tonin et al.^44^ exemplified this by integrating *operando* XTM with *operando* spatially resolved XRD studies on Li–S cells. Their study revealed the transformation of sulfur into smaller, sparsely distributed β-sulfur particles during discharge, which preferentially deposited on the electrode surface. Upon recharging, sulfur crystallized as monoclinic β-S_8_, resulting in smaller particles with a narrower size distribution, impacting electrode morphology and performance.

In their subsequent study,^45^ a new cell design was employed to combine X-ray absorption tomography and XRD computed tomography. Cycling at a C/20 rate, they observed that, when sulfur was reduced to soluble polysulfide during discharge, it led to a nearly 80% collapse in the sulfur–carbon binder domain thickness while the nonwoven carbon binder layer remained intact. This substantially decreases electrode thickness and affects electronic and ionic pathways, ultimately impacting the overall battery performance.

A recent study by Sadd et al.^40^ utilized a combination of *operando* XTM and optical imaging within a capillary cell to study the conversion, dissolution, and precipitation processes in the cathode, as well as the diffusion of LiPSs from the cathode to the bulk electrolyte. Their cell design allowed all components to remain within the field of view while maintaining a small pixel size of 325 μm. This enabled precise tracking of each part, revealing that all elemental sulfur in the cathode was converted, and the resulting products dissolved in the electrolyte. They observed uniform deposition of Li_2_S with particle sizes smaller than 1 μm. The precipitates covered the entire electrode surface, blocking further conversion of LiPSs still present in the electrolyte. Importantly, they suggested that the initial morphology of the S/C composite is not significant because the final precipitation occurs uniformly rather than only in the pores formed by the dissolving elemental sulfur particles.

Overall, the advancements in X-ray imaging techniques have significantly enhanced our understanding of the morphological evolution of sulfur cathodes. These methodologies collectively demonstrate that *in situ* and *operando* X-ray imaging, radiography, and tomography are invaluable for elucidating the complex dynamics within sulfur cathodes, guiding the development of more efficient and durable sulfur-based batteries.

### Neutron Imaging (NI) - 2D Radiography and 3D Tomography

In the previous section, we discussed how X-ray imaging techniques have been extensively used to study sulfur-based batteries. However, there is growing interest in employing NI^46^ to gain new insights into the behavior of the S cathodes during the charging and discharging cycles.

Neutron-based techniques complement X-ray methods due to their distinct interaction with matter. Unlike X-rays, neutrons are highly sensitive to the properties of nuclei rather than electron density, making them particularly effective for detecting light elements such as hydrogen and lithium, which are challenging to observe using X-rays. Neutron techniques also take advantage of isotope differences, enabling researchers to track various components and processes within a sample by substituting isotopes.

NI has great potential in battery research. This nondestructive technique enables direct visualization of lithium diffusion^47^ and provides unique quantitative insights into lithium distribution within the cathode material. In particular, *in situ* neutron tomography creates 3D images of lithium distribution at different states of charge (SoC), while 2D radiography in an *operando* setting captures dynamic changes in lithium distribution during lithiation and delithiation.^48^

NI is slower and has lower spatial resolution than X-ray imaging. This lower spatial resolution, typically ranging from 5 to 100 μm,^49^ makes it suitable for the relatively fast measurements required for *operando* studies. Recent advancements in high-resolution NI have significantly enhanced its capabilities, reducing pixel sizes to 1.5 μm and enabling detail resolution at around 4 μm.^50^ These developments are unlocking new possibilities for studying sulfur-based batteries, where the unique contrast provided by neutrons can yield fresh and complementary insights into systems characterized by high-volume exchange and complex conversion reactions.

As already noted, one of the key advantages of NI is the sensitivity of neutrons to certain low atomic number (low-Z) materials. This is in contrast to X-rays, which are more sensitive to high-Z materials. The effectiveness of NI depends significantly on the neutron attenuation cross sections of the materials in the battery studied. The challenge in sulfur-based batteries is that often these cross sections are similar to those of the bulk components making NI less effective, therefore high-resolution X-ray CT is often a better choice in such cases.

Another advantage of NI is that electrically neutral neutrons interact more weakly with the matter, allowing for greater penetration depth than X-rays. This makes NI particularly effective when material composition and penetration depth are more critical than achieving high spatial resolution.

During NI measurement, the sample is placed in front of a thermal or cold neutron beam, and a detector records the attenuation of the beam as it passes through the sample. The transmitted intensity follows Lambert–Beer’s law, meaning that the ratio of transmitted to incident beam intensity is correlated exponentially with the sample’s thickness and its attenuation coefficient. For 3D neutron tomography, 2D radiographs are recorded while the sample rotates. 3D neutron tomography is more time-consuming and thus typically performed *ex situ* or *in situ* rather than *operando*.^51,52^ However, it can be complemented with *operando* 2D neutron radiography to provide information on species distribution and morphology within the battery.

**Figure 4:**
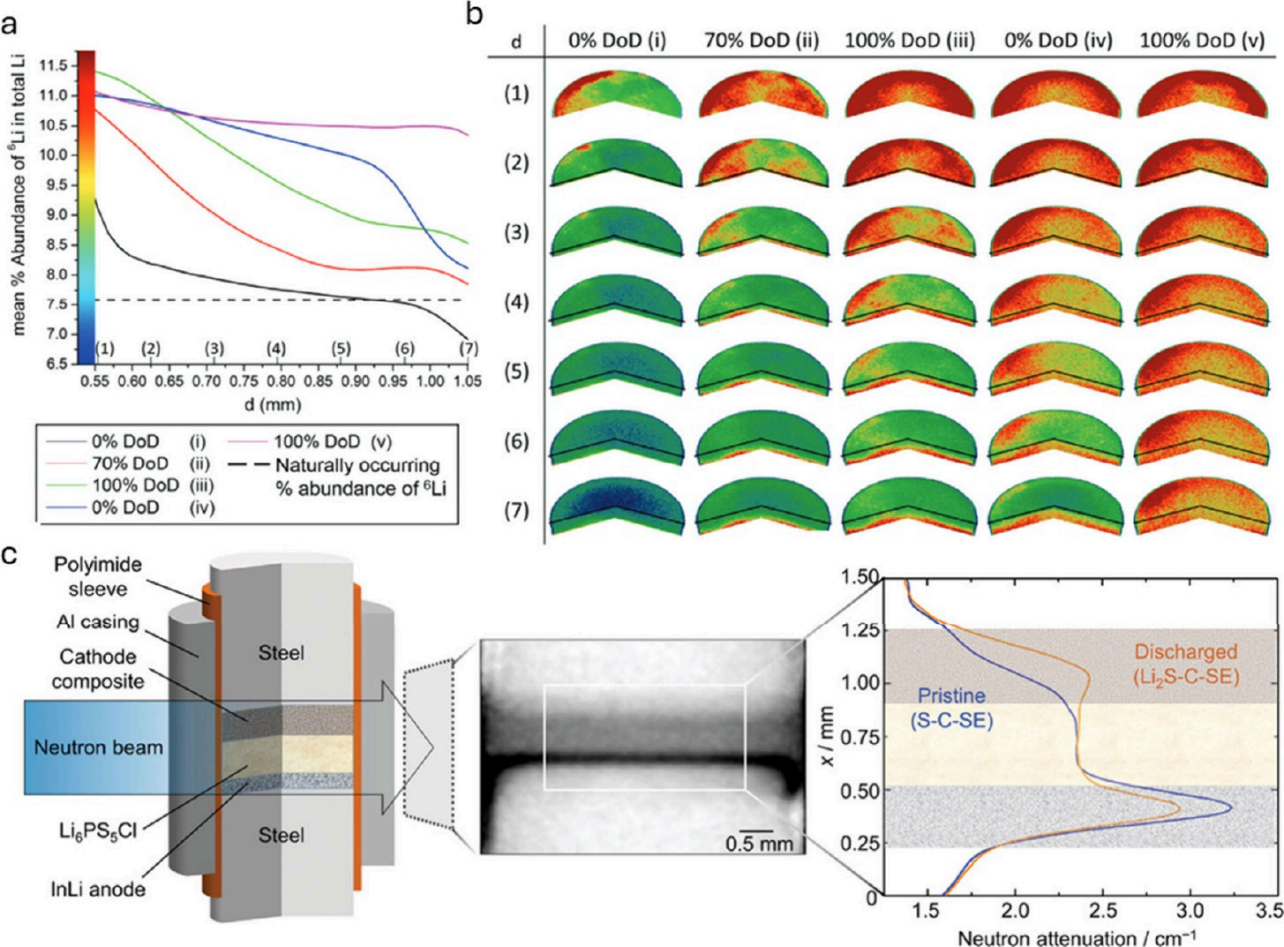
Recent *in situ* NI studies of LSBs, including setups and results. (a) Plot showing the mean percentage abundance of the ^6^Li isotope in the lithium contained within the solid electrolyte separator region. (b) 3D representations showing the inhomogeneity of the percentage abundance of the ^6^Li isotope, for each of the charge states shown in (a). Reproduced with permission from ref (53). Copyright 2023, Wiley-VCH. (c) *In situ* cell design, representative neutron radiography, and neutron attenuation for LSBs. Reproduced with permission from ref (54). Copyright 2023, Wiley-VCH.

Attenuation can be tuned by selective isotope enhancement. For instance, natural lithium consists of two isotopes, ^7^Li (92.58%) and ^6^Li (7.42%). However, the ^7^Li is a weak neutron absorber while ^6^Li is a strong one, with neutron attenuation coefficients Σ of 0.067 and 78.313 cm^–1^ for ^7^Li and ^6^Li, respectively, using cold neutrons with a 2.8 Å wavelength.^50^ Using lithium isotopes as contrast agents makes NI particularly useful for studying the lithium diffusion and intercalation dynamics in rechargeable cells. By substituting isotopes, the contrast of different components within the cell can be manipulated. For instance, NI can reveal the presence of lithium that remains trapped within the cathode composite in the form of Li_2_S, which cannot be reconverted to elemental sulfur (S_8_). This trapped lithium is particularly concentrated near the current collector side of the cathode (Figure 4a,b).^53^ A recent study by Bradbury et al.^54^ on lithium-thiophosphate-based SS LSBs (In/Li | Li_6_PS_5_Cl | S/C/Li_6_PS_5_Cl) marked the first *operando* NI analysis on a sulfur-based SS battery (Figure 4c). The authors enhanced ^6^Li content in the anode while using natural lithium in the SS electrolyte and cathode, making the lithium in the anode the highest neutron absorber among the elements in the cell (excluding indium, which remains immobile during electrochemical operation). This setup allowed a direct correlation between changes in neutron radiography contrast and shifts in lithium concentration. By combining 2D *operando* neutron radiography, which captured the dynamics of reaction propagation, with 3D *in situ* tomography, they gained insights into the spatial distribution of residual lithium. A key finding was identifying a reaction front propagating from the separator toward the current collector during discharge, attributed to sluggish ionic transport in the cathode composite, which led to a persistent lithium gradient even after recharging. Through *in situ*/operando neutron imaging/tomography, the behavior of Li ion transport and the distribution of electrolyte isotopes can function as contrast enhancements or tracers; the technique facilitates the monitoring of gas development.^53^

### Small-Angle X-ray and Neutron Scattering (SAXS/SANS)

Noninvasive small-angle scattering (SAS) techniques, such as SAXS and SANS, are well-established for studying porous materials.^55−57^ The fundamental concept and theory behind X-rays and neutrons are similar. Both techniques offer high spatial resolution on short time scales, with *quasi-operando* SANS providing better contrast and *operando* SAXS offering superior time resolution.

SAS techniques are particularly well-suited for analyzing complex nano- and microstructures, providing statistically averaged information on structure rather than real-space pictures of particular instances.^58^ These methods provide crucial information on surface area and distribution of particle and pore shapes and sizes, ranging from subnm to 100 nm. This data offers critical insights into the relationship between the structure and properties of battery materials.^59^ However, it is important to note that the data obtained is averaged over the sampled volume, making it difficult to draw conclusions about open or closed pores without proper contrast. When employing operando characterization methods, it is vital to consider the risk of radiation damage, particularly at high-flux sources like synchrotrons. Neutrons offer greater penetration compared to X-rays, making it possible to work with standard battery cells, such as pouch or coin cells. However, thick samples may encounter issues with multiple scattering, which can result in inaccurate data interpretation.

SAXS and SANS have been effectively applied to study sulfur-based batteries within electrochemical cells both *in situ* and under *operando* conditions.^60−62^ In LSBs, SAS is often used to investigate carbon host materials in the sulfur cathode, particularly the location of sulfur within the structure. SAS profiles provide valuable information on the chemical composition at the carbon matrix surface, indicated by variations in scattering intensity and slope. Due to the similar electron density of sulfur and carbon, resulting in low contrast at the sulfur–carbon interface,^63^ SAXS is mainly used to probe the structure of sulfur-filled carbon pores by comparing samples at different sulfur loadings, where the signal from empty pores of a specific size diminishes once filled with sulfur.

Like XRD, SANS and SAXS are ineffective in detecting long- and short-chain polysulfides within the electrolyte. However, the complementary use of these techniques enables the investigation of the complex structural interplay between the porous carbon network and the sulfur species in sulfur-based batteries.

Smorgonskaya et al.^64^ were the first to apply SAXS to study carbon/sulfur systems. After introducing sulfur into the carbon matrix, they observed that the scattering intensity decreased nonuniformly, indicating that not all the pores were filled with sulfur. By further analyzing SAXS profiles using the Guinier approximation, they determined the size distribution of sulfur particles and confirmed that the smallest nanopores (8–16 Å) remained empty. Since then, the impact of sulfur loading on its infiltration in carbon host materials has been explored with both SAXS and SANS in several other works. Numerous studies have agreed that pore filling in these host materials strongly depends on the sulfur loading.^59,61,65^

*Quasi-operando* SANS studies enable the characterization of microstructural changes in sulfur-based batteries during galvanostatic charge and discharge, with interruptions for EIS measurements, as demonstrated by Risse et al.^60^ In this work, contrast matching of the carbon matrix with deuterated electrolyte was used to enhance sensitivity to sulfur and lithium sulfide phases. In the SANS pattern, a shoulder appeared at about 2 nm^–1^ during discharge, correlating with the precipitation of Li_2_S in the nanoscopic structures. During galvanostatic charging of LSB, sulfur formed large structures that did not produce significant scattering intensity within the observed length scale. The results indicated that neither sulfur nor Li_2_S was deposited in the micropores during the experiment. Further insights were provided by *operando* SAS studies by Chien et al., who used different contrast conditions to decipher the different scattering contributions.^63^ The analysis suggests that the precipitates are small and unlikely to block the pores of the carbon matrix, which is critical for maintaining battery performance. At C/10 and C/20 discharge rates, a strong correlation between the end of discharge and the change in contrast of the larger spheres indicates a Li^+^ deficiency in the electrolyte within the carbon matrix mesopores.

These results are consistent with the precipitation of Li_2_S_2_ from solution and subsequent conversion to solid Li_2_S crystals.^62^ However, the observed Li_2_S/Li_2_S_2_ composite structure is inconsistent with a stepwise electroreduction of LiPSs at the carbon-electrolyte interface. Instead, the formed structural features suggest that species in the electrolyte favor the growth of the structure. One possibility is that Li_2_S dissolves into Li^+^ and S^2–^ ions and then precipitates again after formation by direct reduction at the carbon. However, due to the low solubility of Li_2_S, the dissolved ions would likely only form small 10 nm Li_2_S crystallites on or near the carbon surface, which quickly form a passivating surface film. Another possibility, the most likely one for the observed superstructures, is the precipitation of Li_2_S_2_. Li_2_S would then form through solid-state electroreduction of Li_2_S_2_. This process would require efficient Li^+^ and e^–^ transport in the solid state. In addition to electroreduction at the carbon-electrolyte interface, Li_2_S_2_ is probably formed by a disproportionation reaction from Li_2_S_4_ in the solution.

The results of *quasi-operando*/ *operando* SANS and SAXS studies have significant implications for the development and optimization of sulfur-based batteries. The incorporation of Li^+^ into the carbon matrix limits sulfur utilization, emphasizing the importance of effective electrolyte management in battery design. The relationship between electrolyte solvation energies and Li_2_S_2_ crystallization affects both the morphology and achievable capacities of LSBs. Proper design of S/Li_2_S structures can facilitate solid-state conversion of sulfur to Li_2_S, avoiding polysulfide shuttling and enhancing battery performance. Despite the growing number of SAS applications in recent publications, the technique is still in its early stages. Given its demonstrated capabilities, *operando* SAS is well-positioned to address technical challenges in battery science, especially those involving time-dependent phenomena at the micro- to nanoscale.

SAS measurements in transmission geometry record scattering curves from all battery components, complicating data interpretation due to overlap. Individual battery components can be measured and subtracted to discern scattering contributions.^60^ Custom cells with only the material of interest in the beam path are used for SAXS, but these alterations may modify chemical processes. SAS data are often featureless, making analysis challenging. Structural changes may produce minor differences in SAS data, complicating data interpretation relying on models constructed from other methods and assumptions about the material’s structural organization. In some cases, analysis is restricted to simple models for limited information extraction, such as slope determination or intensity changes.^66^ The practicality of employing *quasi-operando* SAS techniques to comprehend battery functionality is well established. Despite its advantages, there is a scarcity of *in situ/operando* SAS studies on batteries focusing on micro- and nanoporous carbon electrodes, particularly in LSB chemistries. Significant areas in battery materials research remain unexplored by SAS. Unlike other methods, a noteworthy challenge in understanding battery function is the solid electrolyte interphase formation, where SAS uniquely offers sensitivity to contrast changes at interfaces. While advantageous over techniques like microscopy that provide only local information, the volume averaging feature of SAS still presents limited sensitivity to battery inhomogeneity.

## Phase and Chemical Analysis

Dynamic monitoring of chemical phases and elemental composition is essential for a comprehensive understanding of the underlying mechanisms and phenomena within the various components of sulfur-based batteries. The evolution of crystalline phases within a sulfur-based battery can be effectively monitored using X-ray diffraction, which detects the regular arrangement of atoms within crystals. Besides, *in situ* techniques commonly used for phase and elemental analysis include XPS, TEM-EELS, and XAS. These methods enable real-time tracking of changes in the valence states, chemical environment, and elemental composition within the different battery components during operation. A particular challenge is the monitoring of liquid polysulfide intermediates, which are typically amorphous and undergo rapid chemical changes. Diffraction or scattering techniques that utilize light sources such as electrons, neutrons, or X-rays are not well-suited to capture these dynamic processes. In these cases, spectroscopy techniques such as UV–vis, Raman, FT-IR, and NMR become invaluable. Additionally, EPR can provide critical information by tracking the coordination of unsaturated bonds in polysulfides, offering unique insights into the complex chemical interactions at play within the battery. This section provides a comprehensive overview of real-time monitoring techniques used to study the evolution of the phases and chemistry of the different battery elements during operation and particularly the behavior of intermediate polysulfides.

### X-ray Diffraction (XRD)

*Operando* XRD enables the real-time probing of crystalline solid phases in batteries, allowing for the monitoring of structural modifications that occur during battery operation under conditions that closely mimic real-world functioning.^67,68^ This technique is sensitive to the formation of metastable intermediates and avoids the risks of sample alteration associated with *ex situ* measurements, making it widely used to study reaction mechanisms, as well as aging and failure processes in batteries.

The first reports on *operando* XRD on sulfur batteries helped establish a consensus on the main structural transformations occurring in LSBs. Nelson et al. reported the gradual dissolution of α-S_8_, with the complete disappearance of crystalline sulfur at the end of the first discharge plateau, followed by its reappearance toward the end of charge.^33^ However, the observation of Li_2_S remained elusive in these initial studies. Later *operando* studies by Cañas et al. and Walus et al. confirmed the formation of nanocrystalline Li_2_S through the lower voltage plateau.^67,69^ The duration of the charge and discharge process during which crystalline S_8_ and Li_2_S can be observed varies across studies due to differences in experimental conditions and analytical techniques, as summarized by Zhang et al.^70^ Despite this, there is consensus that during charging, sulfur recrystallizes into the high-temperature monoclinic β-S_8_ allotrope, which is kinetically favored over the thermodynamically stable α phase.^71^ In the subsequent cycles, sulfur continues to recrystallize in the β- S_8_ form.

While XRD is a priori unsuitable for probing the formation of LiPSs due to their solubility, Conder et al. reported observing the evolution of two broad peaks related to high-order LiPSs, such as Li_2_S_8_ and Li_2_S_6_, when using glass fiber separator in which the fumed SiO_2_ acts as a polysulfide scavenger.^72^ Additionally, Paolella et al. identified crystalline Li_2_S_2_ as a transient species formed not as a reaction intermediate but through a disproportionation reaction from higher-order polysulfides under specific conditions, such as highly concentrated “solvent-in-salt” electrolytes.^73^ However, it has been experimentally proved, by synthetically making different Li_2_S_*n*_ polysulfides, that α-S_8_ and Li_2_S are the only stable crystalline phases. *In situ* and *operando* XRD have also been employed to investigate the formation of crystalline deposits on the Li metal anode resulting from the shuttling of Li_2_S_*n*_ species and electrolyte decomposition, and to study the benefits of strategies such as the use of polymer interfacial layers to prevent the electrolyte decomposition at the Li electrode.^74^

Beyond LSBs, *operando* XRD has been utilized to study reaction mechanisms for other monovalent ions, such as Na and K. Unlike LiPSs, several Na_2_S_*x*_ compounds are thermodynamically stable at room temperature, resulting in a wider variety of crystalline sodium polysulfides detectable by *operando* XRD. In Na–S batteries, during discharge, the characteristic peaks of S_8_ fade away, giving rise to long-chain sodium polysulfide Na_2_S_4_ peaks. As discharge progresses, short-chain polysulfide Na_2_S_2_ forms, followed by Na_2_S at the end of discharge. During subsequent charging, the reverse process (Na_2_S → Na_2_S_2_ → Na_2_S_4_ → S_8_) is observed.^75,76^ Additionally, Na_2_S_5_ has been detected during the early stages of discharge.^77,78^ In contrast, K–S batteries have been less extensively studied via *operando* XRD. Similarly to LSBs and Na–S batteries, S_8_ is detected at the initial and final discharge and charge stages, with K_2_S observed as the final reduction product. During the intermediate stages, several potassium polysulfides (K_2_S_6_, K_2_S_4_, K_2_S_3_, and K_2_S_2_) are formed through combinations of precipitation from the liquid and direct solid–solid conversion.^3,79^ For emerging divalent metal–sulfur batteries (Mg^2+^ and Ca^2+^) reports on *operando* XRD are still scarce. However, *in situ* XRD has been used to investigate Ag catalysts in Mg/S cells, revealing the formation of an AgCl layer that prevents the poorly reversible MgS from forming.^80^

In this last direction, catalysts are widely used in advanced cathodes to accelerate the redox reactions of sulfur. The evolution of the catalyst structure and the underlying catalysis mechanisms are critical aspects that can also be effectively monitored using *operando* XRD, providing valuable insights into their role in enhancing battery performance. Both laboratory and synchrotron-based *operando* XRD have become widespread characterization techniques to investigate reaction mechanisms in catalytic cathodes. *Operando* XRD has also been employed to study various catalysts as well as catalyst optimization strategies like surface engineering and defect engineering,^81^ all aimed at promoting polysulfide conversion. Additionally, different nucleation behaviors were observed on open-hollow S decorated with MnO_2_ nanosheets, leading to faster Li_2_S and β-S_8_ nucleation during discharge and charge processes.^82^

Due to the complex reaction mechanisms in metal sulfur batteries, which involve not only structural transformations of crystalline compounds but also the formation of amorphous and liquid intermediate species, combining multiple *in situ* and *operando* techniques has proven essential for a detailed understanding of this chemical system. The combination of *operando* XRD with *operando* XAS,^68^ Raman spectroscopy,^83^ and others has proven highly effective for probing both liquid and solid transformations. Additionally, coupling diffraction techniques with *operando* imaging methods, such as combining XRD and XTM, enables visualization of both the morphological and crystal structure evolution of materials. This allows researchers to monitor the state of the active material throughout the entire cell and correlate it with electrochemical performance.^44,84^

### X-ray Photoelectron Spectroscopy (XPS)

XPS is a widely used technique for studying the surface chemistry of battery materials, particularly sulfur-based materials.^85^ XPS is highly surface sensitive, with a probe depth of only a few nanometers when using X-rays in laboratory settings and up to ∼20 nm when synchrotron X-rays are employed. It typically requires an ultrahigh vacuum (UHV, <10^–7^ Pa) environment to avoid interactions between photoelectrons and gas molecules, which complicates the study of batteries under *operando* or even *in situ* conditions.

*Ex situ* XPS has been used to unveil the intermediate phases and track sulfur redox reactions both in LSBs^86,87^ and post-Li sulfur-based batteries, such as K–S,^3^ Mg–S,^88^ and Al–S batteries,^89^ as well as to study the conversion mechanisms of transitional metal sulfides like MoS_2_,^90^ MoS_3_,^91^ and VS_4_^92^ in batteries. Additionally, *in situ*/*operando* XPS has been applied to investigate Li-electrolyte interfaces in batteries with both liquid and solid electrolytes.^93,94^ However, the application of *in situ* or *operando* XPS in sulfur-based batteries presents significant challenges, and only a few works have been published. This is largely due to the volatility of organic liquid electrolytes, the problematic sublimation of sulfur under typical UHV conditions, and the solubility of intermediate polysulfides in liquid electrolytes.

**Figure 5:**
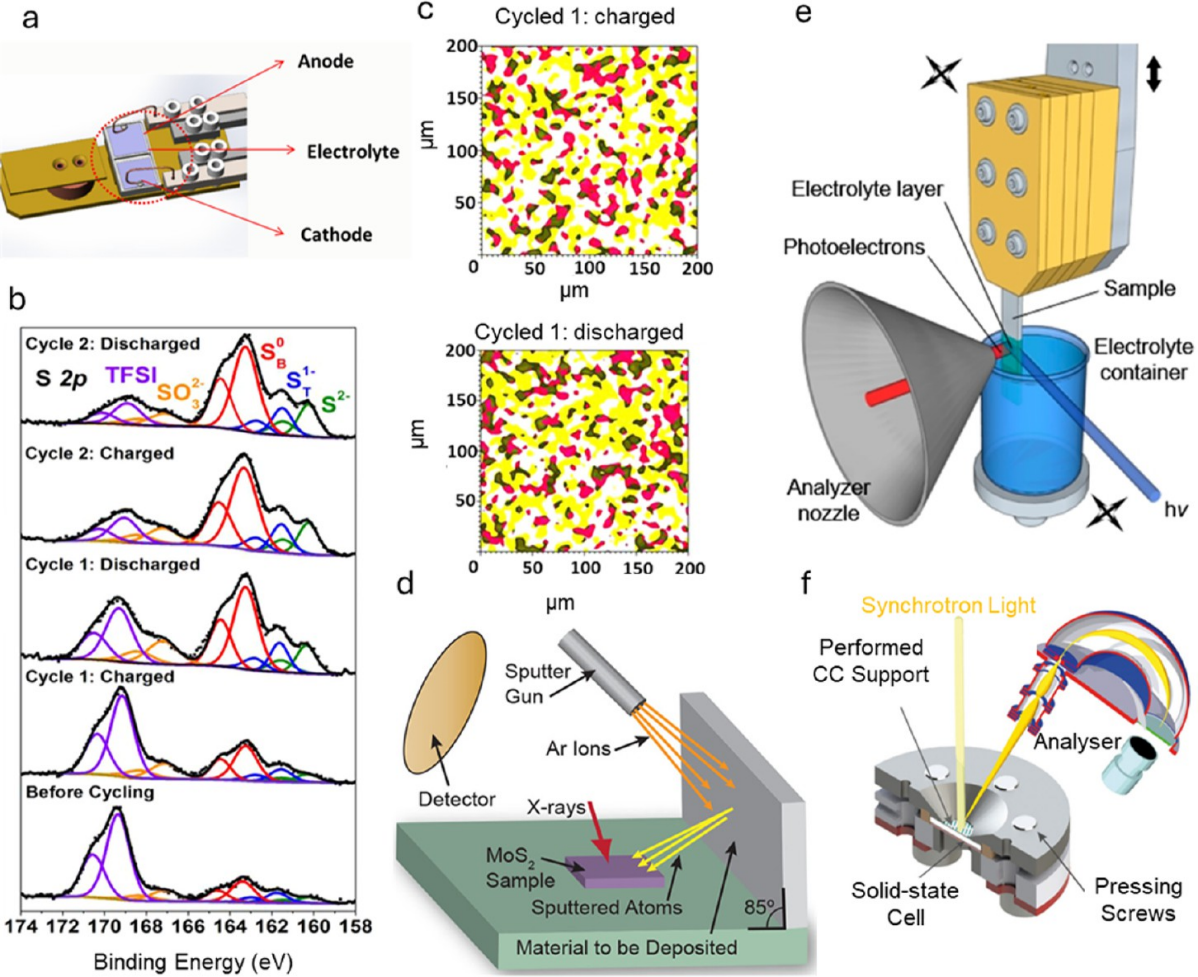
(a–c) *In situ* XPS of the Li-electrolyte interfacial region of LSB at the charged and discharged states. (a) Schematic of *in situ* XPS cell. (b) Core level S 2p XPS spectra. (c) 2D XPS chemical imaging. The red and yellow regions represent Li–F species from F 1s spectra and S^0^ polysulfide species from S 2p spectra, respectively. Their overlapping regions are in black. Reproduced with permission from ref (95). Copyright 2017, American Chemical Society. (d) *In situ* XPS experimental setup of MoS_2_ upon sequential Li deposition. Reproduced with permission from ref (96). Copyright 2017, American Chemical Society. (e) “Dip and pull” *in situ* NAP-XPS setup for studying solid–liquid interfaces. Reproduced with permission from ref (98). Copyright 2019, Elsevier. (f) *In situ* XPS for studying solid–solid interfaces in solid-state batteries. Reproduced with permission from ref (94). Copyright 2024, American Chemical Society.

The first application of *in situ* XPS in LSB research was reported by Murugesan et al.^95^ To address the evaporation issue of liquid organic electrolytes, a 1-butyl-1-methylpyrrolidinium bis(trifloromethylsulfonyl)imide ([bmpyr]^+^[TFSI]^−^) ionic liquid-based electrolyte, with a very low vapor pressure (∼10^–10^ Pa) compatible with UHV environment, was used. The *in situ* XPS cell used in this study is shown in Figure 5a. XPS spectra were acquired at the Li-IL electrolyte interfacial region in both the charged and discharged states. High-resolution core-level S 2p XPS spectra (Figure 5b) showed a gradual increase of sulfide (S^2–^), associated with the formation of insoluble Li_2_S, and a simultaneous increase of polysulfide species (S^0^ and S^1–^) at both charged and discharged states. The authors also performed 2D chemical imaging at charged and discharged states (Figure 5c), unveiling the clustering of reactive solutes, such as polysulfide and Li–F-related species, at the Li-IL electrolyte interfacial region that participated in the SEI formation. In another study, McDowell et al.^96^ used *in situ* XPS to investigate the phase transformation of MoS_2_ during Li deposition, replicating the Li-ion intercalation process seen in MoS_2_-based Li-ion batteries. In this study, the XPS spectra were acquired after sequentially sputtering Li onto MoS_2_ (Figure 5d). By comparing the XPS spectra before and after Li deposition, it was determined that Li reacts readily with MoS_2_ to form metallic Mo and Li_2_S.

To fully harness the potential of XPS for analyzing electrochemical processes in sulfur-based batteries, significant technical advancements are necessary, including the development of new *in situ* XPS techniques and dedicated experimental setups, including *in situ* cells. These improvements should focus on:

(1) To enhance probe depth, high-energy synchrotron radiation-based hard X-ray photoelectron spectroscopy (HAXPES)^97^ has attracted great interest in battery research. However, the use of high-energy X-rays increases the radiation damage, such as alterations in surface chemistry due to X-ray exposure or high kinetic energy secondary electrons.
(2) To overcome the limitations of UHV conditions and address challenges related to the volatility of liquid electrolytes, near ambient pressure (NAP)-XPS has emerged as a powerful technique for investigating electrochemical processes at solid–liquid interfaces under controlled environmental conditions. A “dip and pull” *in situ* NAP-XPS setup^98^ (Figure 5e) creates a liquid layer of nanometer thickness, enabling the study of solid–liquid interfaces, which is promising for sulfur-based battery research. However, current NAP-XPS systems operate at a pressure of 10–20 mbar, which is orders of magnitude below the ambient operating conditions of sulfur-based batteries. Additionally, NAP-XPS requires a synchrotron light source to provide high-intensity photons, which has limited availability and thus restricts its use in *real-time* characterization of sulfur-based battery materials during electrochemical operation.
(3) Solid–solid electrochemical interfaces in solid-state sulfur-based batteries are usually buried and inaccessible. A dedicated experimental setup, such as a UHV-compatible *in situ* XPS cell (Figure 5f),^94^ is necessary to expose the surface/interface of interest for XPS analysis.
(4) To avoid sulfur sublimation under UHV and suppress radiation damage, particularly in HAXPES, cooling the sample, such as with liquid nitrogen, is an effective approach. Recently, cryo-XPS has been proven to suppress radiation damage, thereby enhancing the accuracy of XPS analysis of LSB materials.^99^ However, *in situ* cryo-XPS of sulfur-based batteries still requires a dedicated cell design.
(5) Last but not least, correlative chemical analysis by coupling *in situ* XPS with other *in situ/operando* spectroscopies, such as XAS, Raman, and FTIR spectroscopy, alongside computational methods, like DFT and MD simulations, is crucial to unravel the redox reactions of sulfur-based batteries.

### UV–Visible (UV–Vis) Spectroscopy

Polysulfides exhibit energy gaps between the highest occupied molecular orbital (HOMO) and the lowest unoccupied molecular orbital (LUMO) within the visible spectrum, enabling UV–vis spectroscopy to distinguish between various polysulfides and measure their concentrations in the electrolyte, where the majority of electrochemical S redox reactions take place.^100^ Additionally, UV–vis allows for the analysis of cathode materials. Furthermore, when coupled with electrochemical measurements, this monitoring of the different dissolved sulfur species this technique facilitates a deeper investigation into the mechanism of sulfur-based batteries.^101,102^

**Figure 6:**
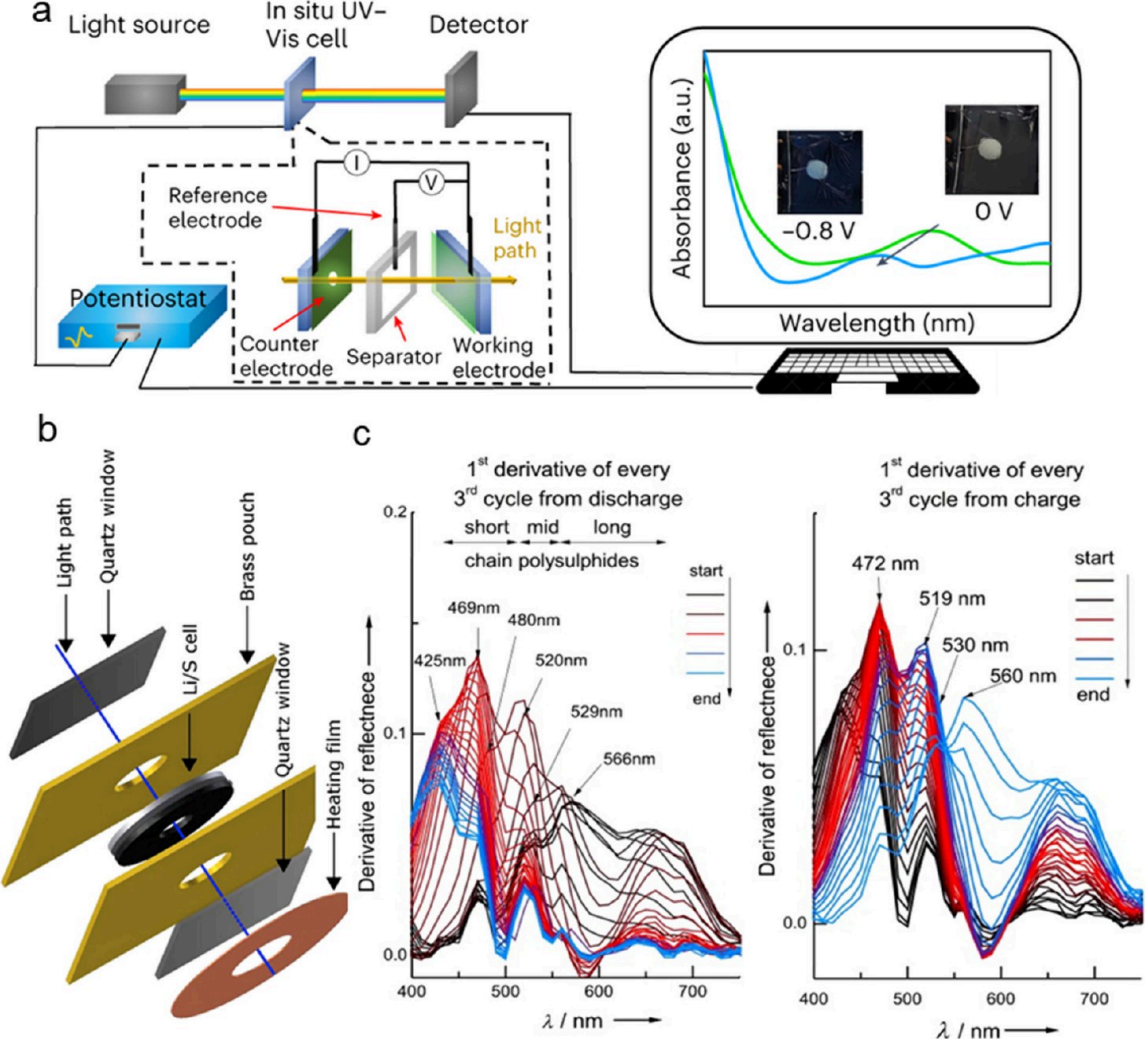
(a, b) Schematic illustration of the *in situ* electrochemical UV–vis spectroscopy setup (a) and cell (b). Reproduced with permission from refs (10) and (103). Copyright 2016, Elsevier and 2023, Springer Nature. (c) Derivatives of UV–vis spectra measured during the discharging and charging of an LSB.^105^ Reproduced with permission from ref (105). Copyright 2013, Wiley-VCH.

Figure 6a illustrates the schematic of the *in situ*/*operando* UV–vis experimental setup.^103^ The analysis of the typically thick and porous cathodes is often performed in reflectance mode, for instance, detecting the dissolution of Mn^2+^ in the intercalation-type materials,^104^ while variations in dissolved polysulfides within the electrolyte are tracked in transmittance mode using a modified device designed with apertures in all the components to allow for light transmission (Figure 6b).^10^

As an example, Patel et al. developed a sealed glass window specifically for pouch cells to perform *in situ* UV–vis measurements.^105^ The setup placed the cell within the path of the incident light beam, enabling reflection mode measurements. From the first-order derivative of the UV–vis spectra, the researchers inferred significant details about the evolution of polysulfide chain lengths during discharge, noting a gradual shortening and an accompanying blue shift in the adsorption peak (Figure 6c). Moreover, UV–vis spectroscopic analysis of the catholyte at varying concentrations revealed that the absorbance at its characteristic wavelength correlated linearly with the natural logarithm of the concentration, as predicted by the Beer–Lambert law, which demonstrates the applicability of *in situ* UV–vis spectroscopy for quantitative analysis method for LSBs. In another study by Zou et al., *in situ* UV–vis spectroscopy was used to examine the electrolyte-dependent redox reaction pathways of S species in LSBs.^106^ The authors conducted a comparative analysis to assess the impact of high-donor-number dimethyl sulfoxide (DMSO) and low-donor-number DME/DOL solvents on LSB performance. Notably, the study revealed that S_3_^•–^ was the most stable intermediate in DMSO-based electrolytes, in contrast to the dominant S_4_^2–^ intermediate in DME/DOL-based systems, highlighting the significant impact of the solvent on the reaction pathways of S species.

UV–vis absorption peaks are highly sensitive to the chemical environment, including variations in cosolvated salt types and concentrations, which translates into notable shifts in the adsorption peaks. For instance, with an increasing LiTFSI concentration in the acetonitrile solvent containing 5 mM Li_2_S_8_, the absorption of S_3_^•-^, S_6_^2–^ and S_8_^2–^ species decreases, while the absorption of S_4_^2–^ increases.^107^ When replacing the 1 M LiTFSI with 1 M tetrabutylammonium bis(trifluoromethanesulfonyl)imide, the S_3_^•–^, S_6_^2–^ and S_8_^2–^ species become dominant.

Future research employing *in situ* UV–vis measurements should focus on standardizing methodologies in sample preparation and normalization procedures, tailored to the electrolyte system under examination. In addition, UV–vis spectra are superimposed absorption spectra, considering that dissolved S species may cause the coexistence of multiple components (typically S_3_^•–^, S_4_^2–^, S_6_^2–^ and S_8_^2–^) in the electrolyte due to chemical equilibria. As a result, when performing *in situ* UV–vis spectra for the reaction mechanism investigation of LSBs, we can try to deconvolve the obtained series of spectra accompanied by their derivatives to enhance the suitability for qualitative and quantitative analyses.

### Raman Spectroscopy

*In situ* Raman spectroscopy has become an invaluable tool in sulfur-based battery research, enabling the detection of shifts in vibrational energy levels, which allows for the monitoring of qualitative changes in polysulfides.^108^ Unlike *in situ* XRD, which is limited to characterizing the crystalline phases of S and Li_2_S, *in situ* Raman spectroscopy is able to detect amorphous and dissolved species due to its sensitivity to molecular bond vibrations. This capability provides a comprehensive understanding of the electrochemical behavior of polysulfides within the Li–S system.^109^

**Figure 7:**
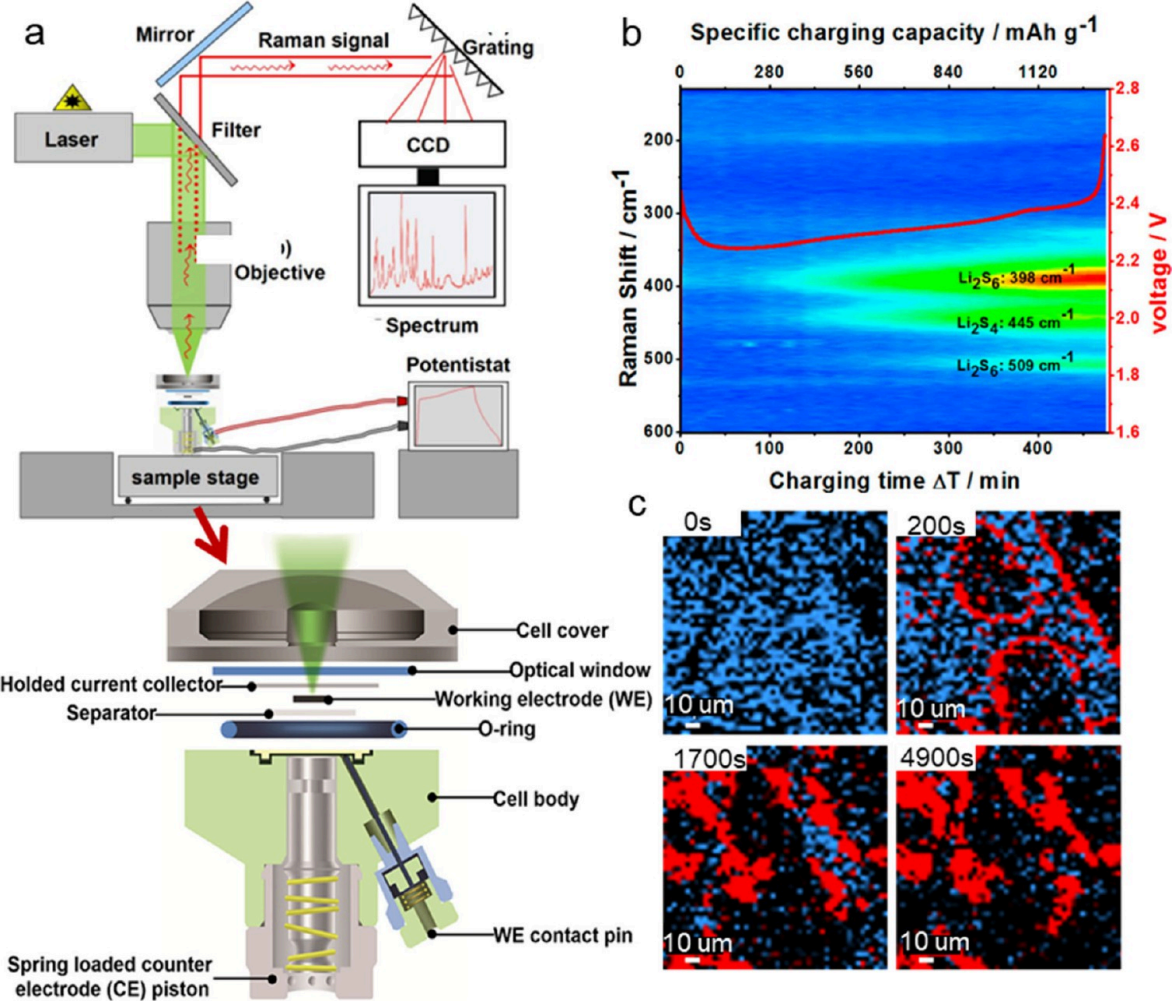
(a) Schematic illustration of the *in situ* electrochemical Raman spectroscopy system and designed device. Reproduced with permission from ref (110). Copyright 2019, American Chemical Society (b) Time sequence of Raman spectra obtained during the 0.1C charging processes of an LSB. Reproduced with permission from ref (111). Copyright 2015, American Chemical Society. (c) *In situ* Raman mapping images during charging and plots of the changes of the polysulfides and S with time. Blue: Polysulfides. Red: S. The color contrasts remain consistent for quantification. Reproduced with permission from ref (113). Copyright 2022, Springer Nature.

The typical setup for *in situ* electrochemical Raman spectroscopy, along with the corresponding cell, is displayed in Figure 7a. This *in situ* cell resembles a coin-type cell but is modified to include a thin quartz window that allows light to penetrate the cell interior. The current collector, separator, and counter electrode are each perforated with a hole, enabling laser illumination and signal collection from the working electrode.^110^

As an example, Chen et al. used *in situ* Raman spectroscopy to monitor the dynamic changes in S species during the discharging and charging of an LSB (Figure 7b).^111^ During the charging process, the final detectable product was Li_2_S_6_, highlighting challenges in achieving the theoretical capacity of LSBs.^112^

Moreover, Raman microscopy offers microscale mapping capabilities that allow the simultaneous characterization of S species at different interfaces. For instance, Lang et al. employed confocal Raman spectroscopy with mapping to track the evolution of S species at the interface of the electrolyte with the conductive cathode network (Figure 7c).^113^ They identified a stepwise S reduction pathway during discharging, contrasted by a parallel oxidation mechanism during charging. Chronoamperometric measurements revealed that both S reduction and polysulfide redox processes follow first-order reaction kinetics, with reaction rates dependent on the electronic conductivity of S particles and the polysulfide concentration. In another study, He et al. examined the distribution of absorbed polysulfides on the surface of a VOPO_4_ polar host.^2^ When dissolved polysulfides adsorb at high coverage on the surface of VOPO_4_, the adsorbed layer acts as a “polysulfide-phobic” interface, repelling dissolved polysulfides in the bulk electrolyte. This repulsion effectively suppresses the shuttle effect, adding a role to the functional interlayer between the cathode and separator.

As a limitation, it is important to note that the Raman characteristic peaks of different S species are consistently below 550 cm^–1^, with each species exhibiting multiple vibrational modes. This overlap of bands can complicate the interpretation and decoupling of Raman spectra.^114^ Additionally, Raman spectroscopy is not able to reliably detect Li_2_S. The characteristic Raman peak of Li_2_S (∼375 cm^–1^) is often either absent or exhibits low intensity, while its crystalline phase can be readily detected by *in situ* XRD from the onset of the lower plateau.^115^

### Nuclear Magnetic Resonance (NMR) Spectroscopy

NMR spectroscopy is a versatile technique for investigating LSBs, with applications including the identification of solid and solvated lithium polysulfides,^112,116−118^ the understanding of polymeric organosulfur cathode materials,^119−122^ and the analysis of ion solvation and clustering in the electrolyte.^123−125^ While *in situ/operando* NMR faces challenges with sensitivity and resolution compared to *ex situ* studies, it remains a highly valuable technique for studying rechargeable batteries.^126,127^ For LSBs, a particular strength of *operando* NMR spectroscopy is its ability to detect both solutions and solids, with no restrictions in size and crystallinity, thus enabling the simultaneous monitoring of the formation of solid *and* solvated polysulfides.

**Figure 8:**
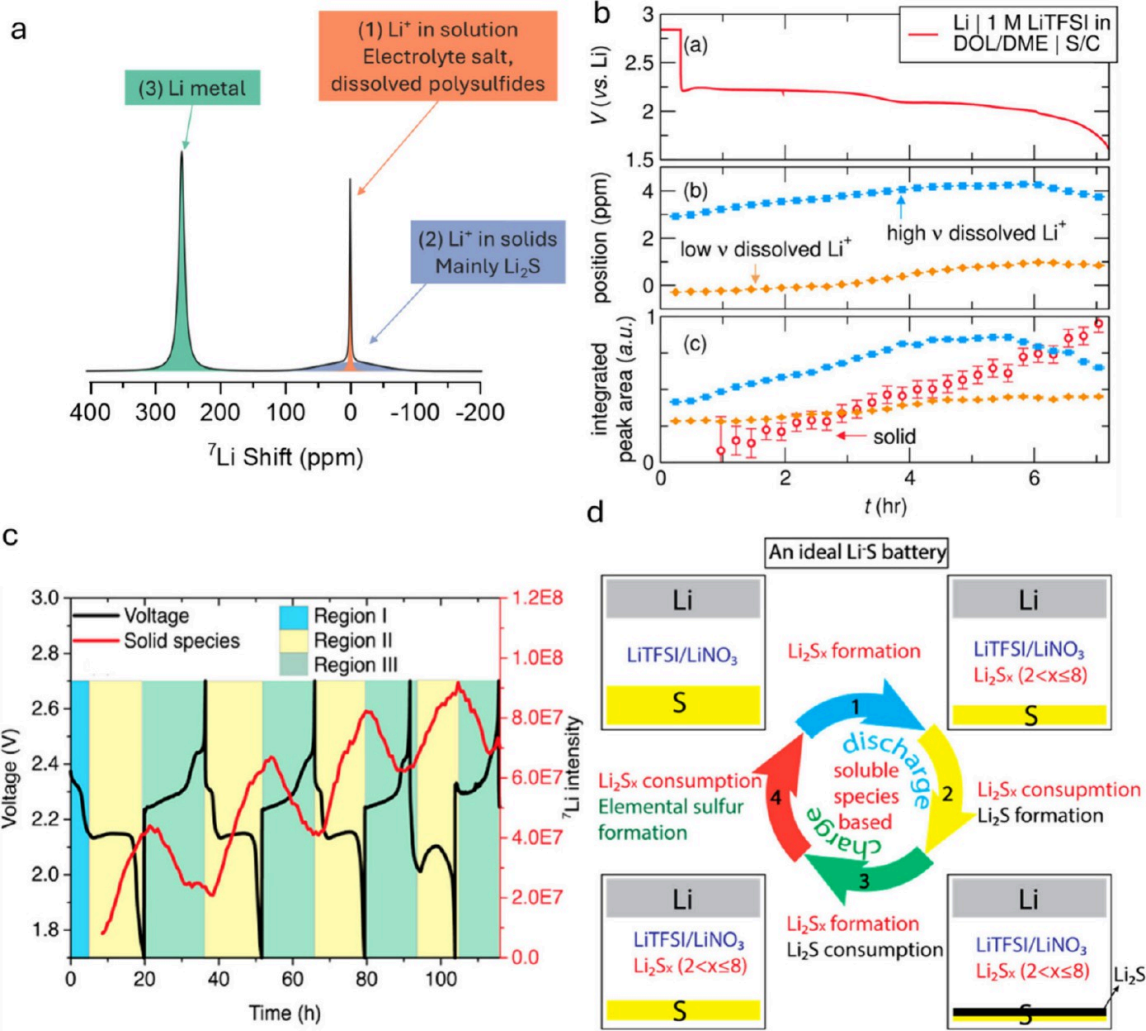
*Operando* NMR of LSBs. (a) Scheme of a typical ^7^Li NMR spectrum of a Li–S cell. (b) ^7^Li *operando* NMR signal deconvolution results and voltage curve obtained from an LSBs pouch cell. Reproduced with permission from ref (117). Copyright 2014, American Chemical Society. (c) Quantity of solid and soluble Li species extracted from ^7^Li *operando* NMR data recorded over four electrochemical cycles and (d) proposed mechanism for an LSB based on this data. Reproduced with permission from ref (130). Copyright 2017, American Chemical Society.

In *operando* NMR studies, ^7^Li NMR spectra are usually recorded due to the high abundance and receptivity of this nucleus. Three groups of resonances can be distinguished in a ^7^Li NMR spectrum of an LSB, as illustrated in Figure 8a.^117^ (1) One or several narrow signals close to 0 ppm corresponding to solvated Li^+^ in the electrolyte from the electrolyte salt and dissolved polysulfides. (2) A broad signal also centered at ∼0 ppm, arising from solid Li salts, mainly Li_2_S.^117^ (3) Signal(s) coming from Li metal, lying between 240 and 270 ppm, where the exact shift depends on the cell orientation and the morphology of Li deposits.^128,129^ While the monitoring of Li metal deposition in different battery chemistries is a very powerful application of *operando* NMR, we focus here on discussing signal groups (1) and (2) that track the sulfur chemistry.

The first *operando*^7^Li NMR experiment on a Li–S cell was reported in 2014 by See et al. on the first discharge recorded in a pouch cell (Figure 8b).^117^ The evolution of signals (1) and (2) during discharge was analyzed, showing that the concentration of Li^+^ ions in solution first increased, indicating the dissolution of polysulfides, followed by a decrease during the second electrochemical plateau, corresponding to the reduction of the dissolved polysulfides to solid Li_2_S. Unexpectedly, solid Li species (mainly Li_2_S) already appeared at the beginning of the first plateau and built up linearly until the end of discharge. It has therefore been proposed that Li_2_S is initially formed by the direct reduction of solid sulfur (first plateau) and later by the reduction of polysulfides in the electrolyte (second plateau).

Wang et al. presented a similar ^7^Li *operando* NMR study on a Li–S pouch cell, reporting a higher number of electrochemical cycles.^130^ Again, the quantity of soluble and solid Li species was tracked based on signals (1) and (2), which led to a description of the polysulfide chemistry as illustrated in (Figure 8c,d): Dissolved polysulfide species are formed during the first electrochemical plateau and are reduced to solid Li_2_S during the second plateau (no Li_2_S appears during the first plateau in this study). During charge, this process is reversed. This mechanism repeats in all cycles, however with a significant irreversible accumulation of solid Li_2_S that reflects the capacity fade of the cell. Additional *ex situ* experiments showed that the majority of Li_2_S was deposited on the anode side, indicating considerable sulfur shuttling at the slow cycling rate used in this study (C/30).

A rather different ^7^Li *operando* NMR study was reported by Xiao et al., using a cylindrical micro-LSB instead of the more common pouch cell format.^131^ Unusually broad and shifted signals were obtained for all regions of the spectrum, hindering the data analysis and the distinction between solid and dissolved species. These effects were attributed to a high concentration of polysulfide radicals as well as the presence of mixed polysulfide species during charge and discharge. However, such large shifts and line widths have not been reported in the other *operando* NMR studies and may also originate from the cylindrical cell format, whose influence on the NMR spectra has not been studied yet.

Dorai et al. introduced an alternative approach to indirectly detect the formation of dissolved polysulfides and radicals in the electrolyte using ^1^H magnetic resonance imaging (MRI), utilizing the fact that the presence of such species changes ^1^H T_1_ relaxation times and hence ^1^H MRI signal intensities.^132^ The use of MRI is attractive because spatial resolution can be obtained, but data interpretation is highly system-dependent due to different trends in ^1^H relaxation times for different electrolyte solvents. In an LSB with a tetraglyme electrolyte, the dissolution of long-chain polysulfides and/or the formation of radical species led to a sharp increase in the MRI signal intensity close to the sulfur cathode, while a subsequent intensity drop to a roughly constant value was explained by the formation of short-chain polysulfides.

To summarize, ^7^Li *operando* NMR experiments of Li–S cells provide valuable insights into the formation of dissolved and solid polysulfide species during cycling as well as the irreversible accumulation of Li_2_S. The results obtained in past studies are conflicting in parts, and it is unclear whether this is coming from different experimental setups and/or from different cell chemistries. Therefore, further studies would be very valuable and could help to unify past results. In addition, *operando* NMR measurements of other nuclei such as ^6^Li and ^33^S in isotopically enriched samples could provide additional insights.^117,118^

### Fourier Transform Infrared (FTIR) Spectroscopy

FTIR spectroscopy is a powerful tool for identifying the chemical bonds and functional groups in organic and inorganic molecules. Monitoring these chemical bond vibrations *in situ*, during electrochemical cycling, allows for further elucidating the interaction between functional materials and LPSs. Analysis can be done both in transmission and reflectance modes on real cells according to the test requirements and real conditions.

**Figure 9:**
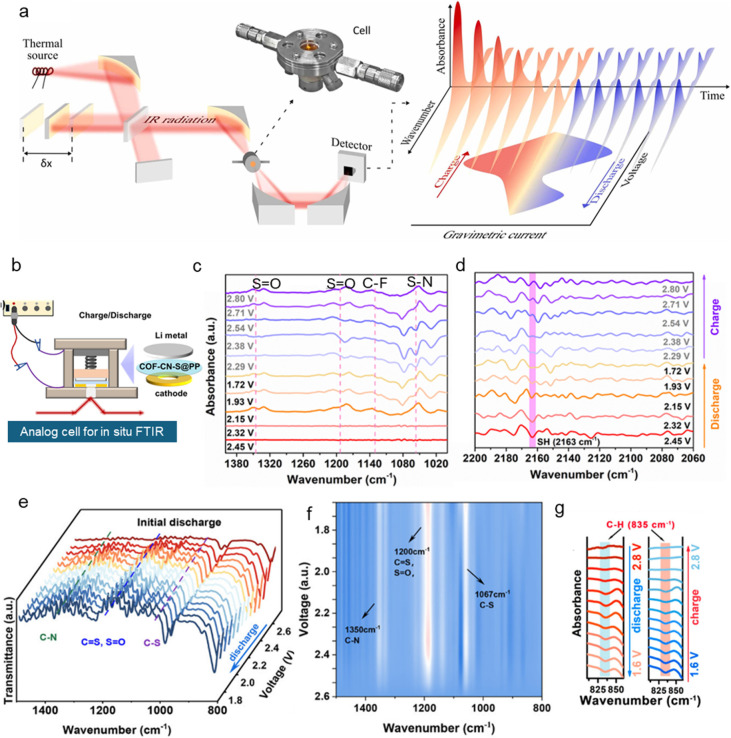
Recent *in situ* FTIR studies of LSBs, including setups and results. (a) Illustration of an *in situ* FTIR setup. Reproduced with permission from ref (134). Copyright 2023, Elsevier (b) Schematic of *in situ* FTIR electrochemical cell. (c, d) FTIR spectra of a COF-CN-S-based battery cycled at a current of 0.1C. Reproduced with permission from ref (133). Copyright 2024, Wiley-VCH. (e) *In situ* FTIR spectra from a COF-SH-modified Li–S cathode and (f) corresponding contour image. Reproduced with permission from ref (137). Copyright 2024, Wiley-VCH. (g) *In situ* FTIR spectra of CNTs-S@G/CTRu electrode monitored in the voltage window 1.6–2.8 V. Reproduced with permission from ref (138). Copyright 2022, American Chemical Society.

As an example, An et al.^133^ engineered a separator coated with a covalent organic framework (COF)-cyanide groups (CN)-S composite, which acted as a cooperative functional promoter to inhibit the shuttle effect and protect both the sulfur cathode and lithium anode in LSBs. The mechanism by which COF inhibits the shuttle effect was examined using *in situ* FTIR. Figure 9a,b depicts the schematic of the battery setup for the *in situ* FTIR apparatus.^134^ As illustrated in Figure 9c, structural changes of the COF-CN-S were monitored across various voltage ranges. The stretching vibrations of the S=O bond in LiTFSI were identified at 1345 and 1193 cm^–1^.^135^ The peaks at 1130 and 1056 cm^–1^, corresponding to the C–F and S–N bonds, remained constant during cycling, indicating an electrostatic interaction between LiTFSI and LiPSs.^136^ Interestingly, the characteristic S–H functional group on COF-CN-S exhibited a dynamic reaction process (Figure 9d). Initially, the S–H vibration showed a concave absorption peak. As charging progressed, the peak intensity gradually increased and shifted from 2163 to 2161 cm^–1^, suggesting that COF-CN-S chemically adsorbs polysulfides through a reversible binding of the S–H (of COF-CN-S) bond to LiPSs, effectively inhibiting the shuttle effect.

Likewise, Bi et al. developed a COF-based quasi-solid electrolyte that exhibited enhanced polysulfide adsorption and conversion capabilities.^137^*In situ* FTIR spectra were acquired to investigate the catalytic performance and the chemical interaction between the COF and Li_2_S_*n*_. Data collection was conducted at the cathode side, with the test voltage ranging from the initial voltage down to 1.7 V. Three distinct peaks were identified at 1067, 1200, and 1350 cm^–1^, which correspond to C–S, C=S/O=S, and C–N bonds, respectively (Figure 9e,f). The intensities of these peaks increased steadily during discharge, indicating that Li_2_S_*n*_ and COF-SH can form strong chemical interactions. These interactions reduce the reaction energy barrier and accelerate the catalytic conversion of Li_2_S_*n*_.

*In situ* FTIR spectroscopy has been also shown valuable to study the impact of catalysts on the evolution of the LiPSs molecular structure. Yang et al. reported a highly efficient Ru-based catalyst graphene/chloro(cyclopentadienyl)bis(triphenylphosphine)ruthenium(II) (G/CTRu) and as an interlayer to capture LiPSs and promote Li^+^ transport in LSBs. To clarify the reaction mechanism, *in situ* FTIR cells were specifically designed and assembled.^138^ The electrode was prepared by coating carbon paper with a CNTs–S@G/CTRu slurry. The LSB was then assembled using the CNTs–S@G/CTRu electrode, a lithium sheet, and a porous Celgard 2400 separator. Figure 9g illustrates the enhancement of the saturated C–H peak at discharge depths of 2.8 to 2.2 V, and then stabilized until 1.6 V. During the discharge, the soluble Li_2_S_4_ eventually changes into insoluble Li_2_S_2_/Li_2_S. Meanwhile, the remaining soluble LiPSs remained adsorbed to the Cp ring through the Li-Cx bond. The subsequent charging process showed signs of reversible evolution. This illustrates that the Cp ring can both donate and accept electrons to and from the LiPSs through the center C atoms, thereby regulating the sulfur reduction reactions.

*In situ* FTIR is a useful technique for tracking chemical bonding vibrations in real-time and examining the relationship between molecular structure evolution and electrochemical properties. However, a key challenge is the strong IR absorption bands of liquid organic electrolytes, which often overlap with signals from active materials, obscuring critical changes occurring at the electrode or electrolyte-electrode interface. Additionally, FTIR faces limitations in detecting low-concentration species within the complex battery environment and lacks the spatial resolution needed to map localized chemical changes, making it difficult to resolve small-scale electrochemical phenomena.

### X-ray Absorption Spectroscopy (XAS)

*Operando* XAS allows for real-time monitoring of the geometric and electronic structures of materials during battery operation, providing crucial insight into chemical bonding, oxidation states, band structures, and local symmetries. This technique offers a detailed picture of the evolving state of materials under working conditions.^139^ As an element-specific technique, XAS is sensitive to the speciation of sulfur in both liquid and solid phases. One of XAS’s key advantages lies in its particular sensitivity to sulfur oxidation states, as sulfur K-edge exhibits a substantial shift of around 6 eV between elemental sulfur and sulfate, allowing for clear discrimination between sulfur species at different stages of oxidation.^140^ Additionally, the shape of the sulfur edge and pre-edge resonances is strongly influenced by the local symmetry of sulfur atoms in the compound, serving as a distinctive fingerprint for identifying individual sulfur compounds in complex mixtures. These capabilities make *operando* XAS an essential tool for elucidating the complex reaction mechanisms, failure pathways, and degradation processes in sulfur-based batteries, especially in tracking polyanionic intermediates during the solid–liquid and liquid–liquid conversion steps.

**Figure 10:**
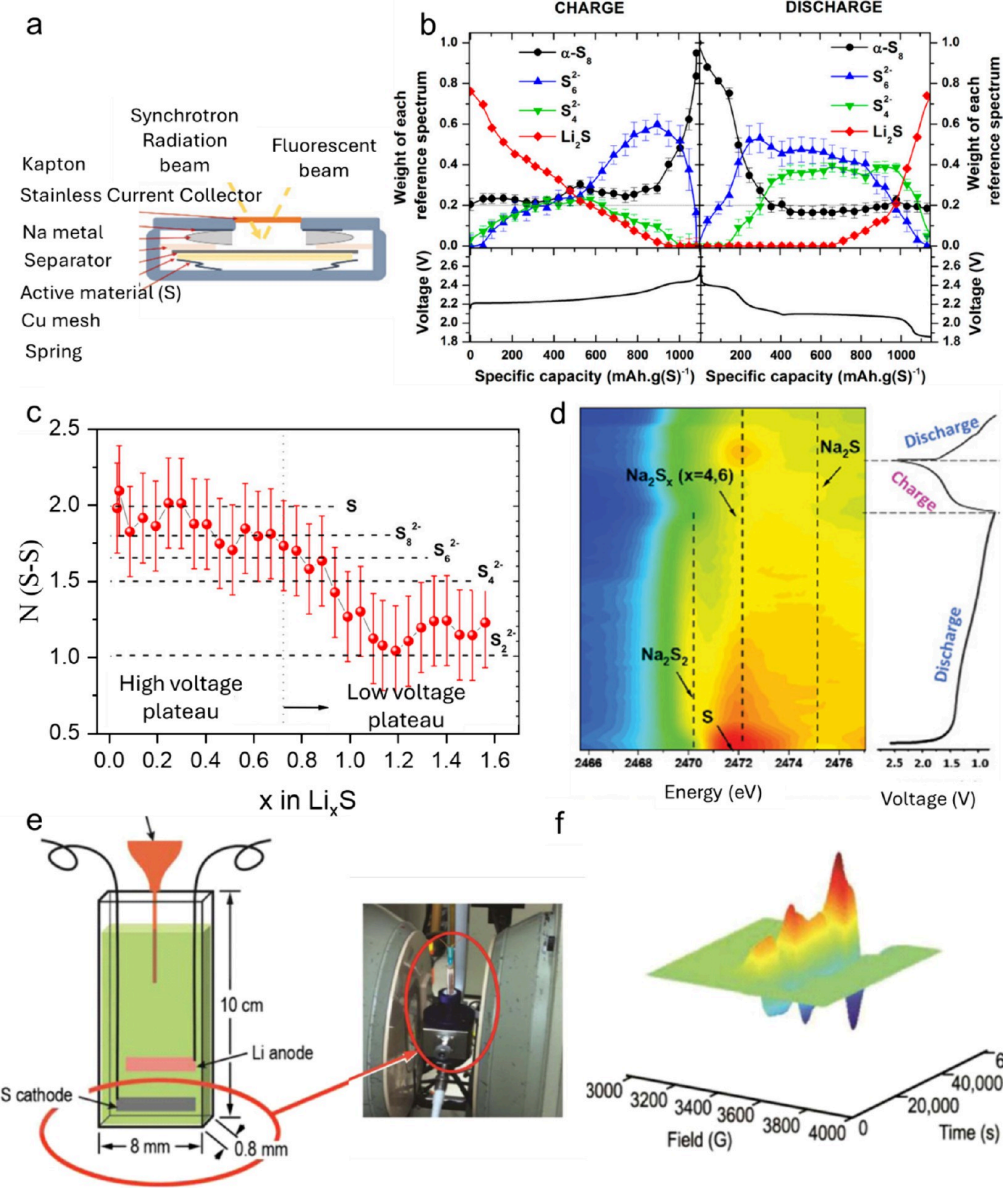
(a) Schematic illustration of an *operando* XAS coin cell used to measure S K-edge in fluorescence mode. Reproduced with permission from ref (141). Copyright 2022, Wiley-VCH (b) Evolution of *operando* sulfur K-edge XANES upon electrochemical cycling based on linear combination analysis. Reproduced with permission from ref (112). Copyright 2013, American Chemical Society. (c) Variation of the average S coordination number during the first discharge. Reproduced with permission from ref (149). Copyright 2015, American Chemical Society. (d) 2D XAS image of different initial S species, intermediates, and final products in an Na–S battery. Reproduced with permission from ref (141). Copyright 2022, Wiley-VCH. (e) Schematics of *in situ* EPR test cell design. (f) 3D plot of *in situ* S_3_^–^ EPR spectra in a functioning Li–S EPR cell vs time during CV scan. Reproduced with permission from ref (175). Copyright 2015, IOP Publishing, Ltd.

The sulfur K-edge falls in the tender X-ray energy range (2–5 keV), which does not strictly require vacuum conditions but does pose some challenges for *operando* cell design due to the limited penetration depth of X-rays at these energies. Thin windows made of lightweight elements are required, typically less than 8 μm thick for Be or Si_3_N_4_, and less than 25 μm for Kapton. These windows are mounted either on modified coin cells (Figure 10a),^141,142^ pouch cells,^143^ or specially designed *operando* cells.^144^ At these tender X-ray energies, it is crucial to minimize absorption by other cell components, namely the contributions from the electrolyte can severely mask those from the sulfur on the electrode. Moreover, due to the low attenuation length, detection is generally limited to total fluorescence yield or total electron yield. An important obstacle in measurements done in fluorescence mode is that they can be heavily distorted by self-absorption effects, generally requiring diluted samples to avoid spectral distortions.^68,72,143,145^ On the other hand, by combining both total fluorescence yield and total electron yield detection modes a chemical depth profile of the electrode can be obtained, ranging from a few nanometers up to several microns, giving a surface and bulk information on the electrode speciation. Another challenge in sulfur K-edge XAS is radiation damage. Prolonged exposure to synchrotron X-rays can induce morphological changes in the sulfur particles, affecting the accuracy of the measurements.^146^ Therefore, experimental conditions must be carefully controlled to minimize such effects.

The first *in situ* XAS experiments on LSBs reported by Gao et al. took advantage of the low penetration depth of S K-edge X-ray radiation to probe the electrolyte phase and track variations in sulfur species across different electrolyte compositions.^142^ Later *operando* XAS studies performed in real-time upon battery operation on Li–S system reported by Cuisinier et al. employed sulfur K-edge X-ray absorption near edge structure (XANES) to follow semiquantitatively the evolution of different sulfur species during the charge and discharge process (Figure 10b). By performing a linear combination fit of the XANES data and comparing it with reference spectra of elemental S_8_ (2472 eV), Li_2_S (2479 eV) and various linear polysulfides (S_*x*_^2–^ with 2 < *x* < 6) (2470 eV), they proposed a reaction mechanism where elemental sulfur is rapidly formed and consumed at expenses of the transient species (S_6_^2–^ and S_4_^2–^) at final and initial stages of charge and discharge, respectively. In contrast, Li_2_S forms rapidly at the end of discharge but is slowly consumed during most of the charging process, revealing a hysteresis related to the different kinetics of dissolution and deposition in carbon–sulfur composite electrodes.^112^

Further *operando* XAS investigations have delivered a detailed molecular understanding of sulfur reaction pathways, especially focusing on speciation and quantification^147^ of polysulfide intermediates.^68,143,144^ The nature of the solvent was found to play a determining role in the solubility of the polysulfide intermediate and the stabilization of free radical species (S_3_^•–^).^145,148^ The analysis of extended X-ray absorption fine structure (EXAFS) spectra has been employed to identify S_8_, Li_2_S, and the determination of polysulfides has been inferred from the variation in the average coordination number of the S–S component (Figure 10c).^149^ The average chain length of sulfur-containing species has also been determined from the ratio of the areas under the main-edge and pre-edge of S K-edge XAS peaks, from which reaction rate constants were estimated.^147^

Highly time-resolved *operando* XAS studies conducted at various C-rates provided evidence for the multistep, kinetic-determining conversion reactions, especially the transition from α-S_8_ to long-chain soluble polysulfides.^150^*Operando* XAS has also been used among other techniques to understand the differences in the reaction mechanism of Li_2_S electrodes compared to S_8_ electrodes.^151^ Moreover, it was used to understand self-discharge phenomena related to the spatial distribution of soluble species^152^ and to assess the various strategies aimed to overcome the current limitation of Li–S technology. These strategies include the selection of optimal electrolytes that minimize the dissolution of polysulfides,^153,154^ methods to reduce the shuttle effect by confining polysulfides using supporting matrices that covalently bond to sulfur,^155^ and the development of multifunctional polymer binders that mitigate shuttling.^156,157^ Further innovations explored using *operando* XAS involve modified separators designed to anchor the polysulfides^158^ and the use of electrocatalysts to reduce shuttling and facilitate the otherwise sluggish polysulfide conversion kinetics.

*Operando* XAS has been also employed to investigate the reaction mechanisms in alternative sulfur-based batteries beyond LSBs, including systems based on monovalent (Na^+^, K^+^) and multivalent (Mg^2+^, Ca^2+^) metals.^159−161^ In these systems, the conversion of sulfur to sulfide generally follows a mechanism similar to that observed in LSBs. *Operando* XAS has enabled the identification of S, metal sulfide (M_2_S), and metal polysulfides (M_2_S_*x*_, where M = Na, K). However, comprehensive mechanistic and quantitative studies on these systems remain scarce (Figure 10d).^141,162,163^ In contrast, more detailed investigations using *operando* XAS on divalent chemistries (Mg^2+^, Ca^2+^) have provided valuable insights into the electrochemical conversion of sulfur into sulfide through polysulfide intermediates. Notably, studies on Ca–S batteries, utilizing principal component analysis (PCA) and multivariate curve resolution-alternating least-squares (MCR-ALS) analysis of sulfur K-edge XANES spectra, have mapped the evolution of polysulfide species, ranging from S_8_^2–^ to S_2_^2–^, revealing a gradual formation of shorter polysulfides as the battery discharges.^164^ Similarly, in Mg–S batteries, linear combination fits performed on *operando* S K-edge XANES spectra have allowed the quantification of four sulfur-containing species (elemental sulfur, MgS_*x*_, electrochemically formed MgS, and electrolyte) within the cathode during the first discharge cycle.^165^ These findings underscore the crucial role of *operando* XAS in unraveling the complex reaction mechanisms in sulfur-based batteries, highlighting its importance for advancing the design and optimization of next-generation energy storage systems.

*Operando* XAS studies are often conducted using a multitechnique approach, combined with other characterization methods such as XRD,^68^ NMR,^143^ UV/vis spectroscopy,^153,166,167^ Raman spectroscopy,^168^ and X-ray fluorescence.^152^ Some studies even integrate several of these techniques simultaneously within a single *operando* cell.^169^

Beyond *operando* XAS, X-ray emission spectroscopy (XES) and resonant inelastic X-ray scattering (RIXS) have emerged as powerful tools for studying LSBs. These techniques provide complementary, element-specific insights into the local electronic structure of sulfur with enhanced resolution and sensitivity, effectively mitigating the core-hole lifetime broadening limitations inherent to XAS. Additionally, self-absorption effects are virtually negligible in these methods. Kα and Kβ XES measurements, in particular, offer high sensitivity to the average charge state of sulfur, which is crucial for determining its oxidation state. *Operando* XES, conducted using a laboratory-scale proton beam, has been employed to monitor the sulfur average charge in real-time, utilizing Kα XES spectra obtained during battery discharge.^170^*Ex situ* valence-to-core Kβ sulfur XES has also been used to quantitatively analyze the electrochemical conversion of sulfur in LSBs.^171^ RIXS, with its heightened sensitivity to LiPSs achieved through resonant excitation, has enabled *operando* quantification of LiPS species in conventional systems with sulfur-containing electrolytes, something otherwise impossible by conventional XANES.^172^ Furthermore, high-energy resolution fluorescence detected XAS (HERFD-XAS) has allowed for the precise identification of Li_2_S_*x*_ polysulfides. In particular, HERFD-XAS accurately resolves the pre-edge peak, attributed to terminal atoms of LiPSs, from the main peak generally associated with elemental sulfur.^173^ Despite their potential, these advanced techniques remain underutilized, primarily due to the limited availability of specialized in-vacuum emission spectrometers required for measurements in the tender X-ray range. Additionally, radiation-induced effects from proton sources pose significant challenges for *operando* measurements, further restricting their application.

### Electron Paramagnetic Resonance (EPR)

EPR is a magnetic resonance technique able to detect species with unpaired electrons, such as free radicals, transition metal ions, and defects. In the field of batteries, EPR is a powerful tool to explore the redox mechanisms, particularly effective in detecting intermediates and transition states in battery reactions. The instability of these intermediates, a common issue in systems like lithium-ion and LSBs, can lead to degraded performance, reduced cycle life, and potential safety risks. *In situ* EPR allows for real-time monitoring of changes in the electronic structure of battery materials, serving as a highly sensitive electronic ’probe’ that reveals the actual operating state of the battery.^4^

Taking advantage of this nondestructive technique, Bai et al.^174^ directly observed the dynamic delithiation evolution of a polyimide electrode during battery cycling through *in situ* EPR. They designed an electrochemical online EPR experiment to detect the redox processes of the electrode in real-time. This setup allowed for the direct observation of a classical redox reaction involving two-electron transfer, evidenced by a distinctive pair of peaks in the cyclic voltammetry (CV) plot, thus providing direct insight into the more complex redox mechanism of LSBs compared to traditional lithium batteries.

In 2015, Wang et al.^175^ conducted *in situ* EPR research on LSB to reveal the different reaction pathways during the cycling processes of LSB. They modified a regular glass cell for *in situ* testing and monitored the generation and concentration variation of sulfur radicals using *in situ* EPR technique (Figure 10e). During the cycling process, S_3_^–^ radicals were consistently detected, indicating a balance among various polysulfides at different potentials rather than the successive formation of a single polysulfide component (Figure 10f). These *in situ* EPR experiments revealed different reaction pathways during discharge and charge. Overall, *in situ* EPR not only provides direct evidence for the evolution of electrodes in batteries but also proves to be an invaluable method for studying complex multielectron reactions within batteries.

## Electrochemical Characterization: Electrochemical Impedance Spectroscopy (EIS)

Several time-domain electrochemical techniques such as CV, chronopotentiometry, chronoamperometry, galvanostatic intermittent titration technique (GITT), and potentiostatic intermittent titration technique (PITT) are critical in the *in situ* study of sulfur-based batteries. In the frequency domain, EIS provides essential insights into the charge-transfer and transport properties of materials. While all these methods introduce an electrical signal within the cell, thereby perturbing its normal operation, they cannot strictly be considered *operando* techniques. However, they are generally applied *in situ* to investigate the electrical and electrochemical properties of the battery components and interphases. Nevertheless, this chapter will specifically focus just on EIS, which is the electrochemical characterization technique most conventionally recognized as an *in situ* tool.

In general, the signal obtained from EIS measurements reflects the combined response of a two-electrode electrochemical system, unless a third or fourth reference electrode is incorporated to enable process separation specific to the anode and cathode. In a two-electrode setup, the EIS signal represents the cumulative contributions from all components within the cell, including the electrodes, separator, electrolyte, and battery casing. This aggregation complicates data interpretation and can obscure contributions directly associated with the active material.

Frequency domain analysis using EIS is a powerful technique for examining the charge-transfer and mass transport events as a function of the SoC of the battery.^176−178^ The effectiveness of EIS largely stems from the application of linear system theory (LST), which significantly aids in the quantitative analysis of batteries. This is accomplished by employing specific models that leverage the principles of LST to interpret complex electrochemical interactions. Generally, the different electrochemical processes present in sulfur-based batteries can be identified by specific time constants (τ). By applying a small sinusoidal potential (δ*E* ≤ 10 mV) or current signal (δ*I* × *R*_ct_ ≥ δ*E* (10 mV)), a linear relationship δ*E* = *Z*(ω) × δ*I* holds, where *Z*(ω) is the frequency-dependent impedance and ω = 2*f* is the angular frequency. This linear relationship is applicable across a range of frequencies, typically from 100 kHz to 10 mHz. The use of small perturbations in electrochemical measurements (quasi-steady state conditions) ensures minimal electrode degradation and significantly simplifies the analysis by circumventing the nonlinear behavior dictated by Butler–Volmer’s kinetic law for electron transfer. Instead, this approach allows for a straightforward linear interpretation, characterized predominantly by the charge-transfer resistance (*R*ct).^179^

Distribution of relaxation times (DRT) analysis, involving the deconvolution of the overlapped time constants, is a particularly powerful approach to interpreting the EIS spectra of sulfur-based batteries.^180,181^ The DRT impedance spectrum is characterized by peaks at various time constants, which can be analyzed to discern different electrochemical phenomena, including (*i*) the S_8_ interparticle resistance, (*ii*) the sulfur–sulfur contact double-layers, (*iii*) the charge-transfer reactions at the anode and cathode, and (*iv*) the mass-transport limitations.^182^

**Figure 11:**
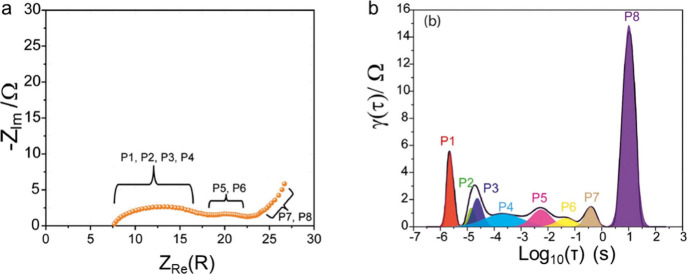
(a) Nyquist plot of the battery with 5 μL/mg sulfur electrolyte recorded at open circuit voltage (OCV) at 100% SoC and (b) DRT plot of the impedance data shown in (a). Reproduced with permission from ref (182). Copyright 2022, Elsevier.

Figure 11a,b displays the typical Nyquist plot of the EIS spectra and the DRT pattern of a two-electrode LSB at 2.6 V. In the Nyquist plot, the depressed semicircle observed at intermediate frequencies is attributed to cathode charge-transfer resistance, while the low-frequency response is indicative of Li^+^ diffusion through the electrolyte. The DRT pattern allows a more accurate appreciation of the data, featuring eight well-defined peaks representing different electrochemical phenomena. The area under these peaks quantifies the relative contribution of the polarization resistance to the overall resistance of the cell. Modifications in the DRT bands reflect changes in the sulfur’s redox reactions, indicating adjustments in electrochemical dynamics.

Additionally, the cathode response in sulfur-based batteries can be analyzed using the transmission line model. In this model, the high-frequency semicircle on the complex-plane plot represents the anode’s charge-transfer resistance, whereas the medium-frequency depressed semicircle is associated with the reduction of sulfur species and the cyclic formation and dissolution of S_8_ and Li_2_S at the cathode.^183^ Further interpretations suggest that the medium-frequency semicircle may also relate to charge-transfer processes coupled with the electrical double-layer structure.^184^ Concurrently, the high-frequency semicircle could represent the particle-to-particle contact resistance involving S_8_ species.

## Gas, Pressure, and Temperature Monitoring

During battery operation, increases in temperature and pressure, as well as gas generation from chemical reactions within the cell, are common. Real-time monitoring of these parameters is crucial for understanding battery performance, optimizing conditions to address related issues, and ensuring safe system operation. This monitoring provides critical insights into the electrochemical processes and degradation mechanisms occurring inside the battery. Moreover, it enables proactive battery management, helping to prevent hazardous conditions such as thermal runaway or excessive pressure buildup. Implementing robust sensor systems for temperature, pressure, and gas detection enhances the overall reliability and safety of battery systems, making it a fundamental aspect of modern battery management strategies.

### Gas Evolution Monitoring

Sulfur-based batteries, like other liquid electrolyte cell chemistries, are particularly susceptible to gas evolution as a result of electrolyte degradation. In LSBs, hydrogen (H_2_) and hydrogen sulfides (H_2_S) are the most common byproducts of parasitic chemical reactions involving sulfur, while hydrogen is a common byproduct of Li anodes.^185^ These and other evolved gases can be effectively identified and monitored *in situ/operando* using a mass spectrometry (MS) system coupled with a properly designed electrochemical cell.^186^ By analyzing the composition of the effluent gases, this technique, often referred to as differential electrochemical mass spectrometry (DEMS), provides real-time insights into the underlying mechanisms of battery operation and degradation. Enhancing this setup with a gas FTIR cell allows for more precise identification of gas molecules generated during cycling. Additionally, the use of isotopically labeled compounds aids in the precise identification of the origin of the evolved gases.^187^ Since gas release is often accompanied by pressure changes within the cell, simultaneous pressure measurements provide a more accurate characterization of battery performance. For example, the first investigation into the gassing behavior of LSBs was reported by Jozwiuk et al.^187^ They monitored the gas evolution of LSBs with a diglyme-based electrolyte, evaluating the effect of the polysulfide shuttle-suppressing additive LiNO_3_ on gas generation. By combining *operando* pressure measurements with DEMS and FTIR analysis, they demonstrated that while the additive significantly reduced gassing, it did not fully eliminate it. The most substantial pressure increase occurred during charging, immediately after fresh lithium deposition. Cells containing LiNO_3_ exhibited evolution of N_2_ and N_2_O, alongside CH_4_ and H_2_, with the latter being the primary volatile decomposition products.

For commercial in-cell detection of gases, the use of metal oxide nanoparticles presents one of the most promising options for several reasons. First, they have been successfully deployed for gas detection, particularly for hydrogen,^188^ demonstrating high sensitivity and selectivity. Second, these nanoparticles have been embedded into prototype cells using glass fiber substrates, showcasing their integration potential.^189,190^ However, their mechanical fragility remains a challenge. Despite this, they offer a unique advantage: being nonconductive, they do not carry current, and instead use optical signals as a data source, minimizing interference with the cell’s electrical performance.

Another approach involves the use of microsurface mount device (SMD) gas sensors based on metal oxides like zinc oxide, which are placed at the transistor gate region to detect gas generation.^191^ However, the main barrier to implementing these sensors is the requirement for gas-permeable membranes to enhance selectivity, which adds complexity and increases the size of the system. In addition, fiber optic sensing technology holds great potential due to its small size, flexibility, and ruggedness, making it compatible with nearly all battery types. This technology could enable fiber optic gas spectroscopy for real-time gas monitoring in rechargeable batteries.^192^

Monitoring gas evolution inside a battery cell can play a crucial role in tracking the state of health, detecting early failures, and understanding the underlying mechanisms of cell degradation.^193^ For instance, a recent study investigating cell failure under thermal runaway conditions analyzed the gas products generated during the process to create a detailed failure map.^194^

In this study, the detection of O_2_ before catastrophic failure signaled the decomposition of the nickel–manganese-cobalt cathode at elevated temperatures, followed by electrolyte decomposition, indicated by the release of CO_2_. The emergence of C_2_H_4_ was attributed to reactions between lithium and the electrolyte, while H_2_ evolved from reactions involving lithium and the binder. Understanding these gas-evolution mechanisms also suggests that monitoring CO_2_ concentration *in situ*, when combined with thermal monitoring, could serve as an early detection mechanism for thermal runaway, enabling proactive management and mitigation of battery failure.

### Pressure Monitoring

The pressure within a battery cell increases not only due to gas evolution but also fluctuates during cycling, reflecting structural changes and volume variations, particularly in the cathode. Particularly in solid-state batteries, applying high external pressure can enhance interfacial contact, improve electrolyte absorption and diffusion, stabilize the lithium–metal anode, and maintain robust connections in the sulfur cathode, thereby preventing cracking throughout the battery’s cycles.

**Figure 12:**
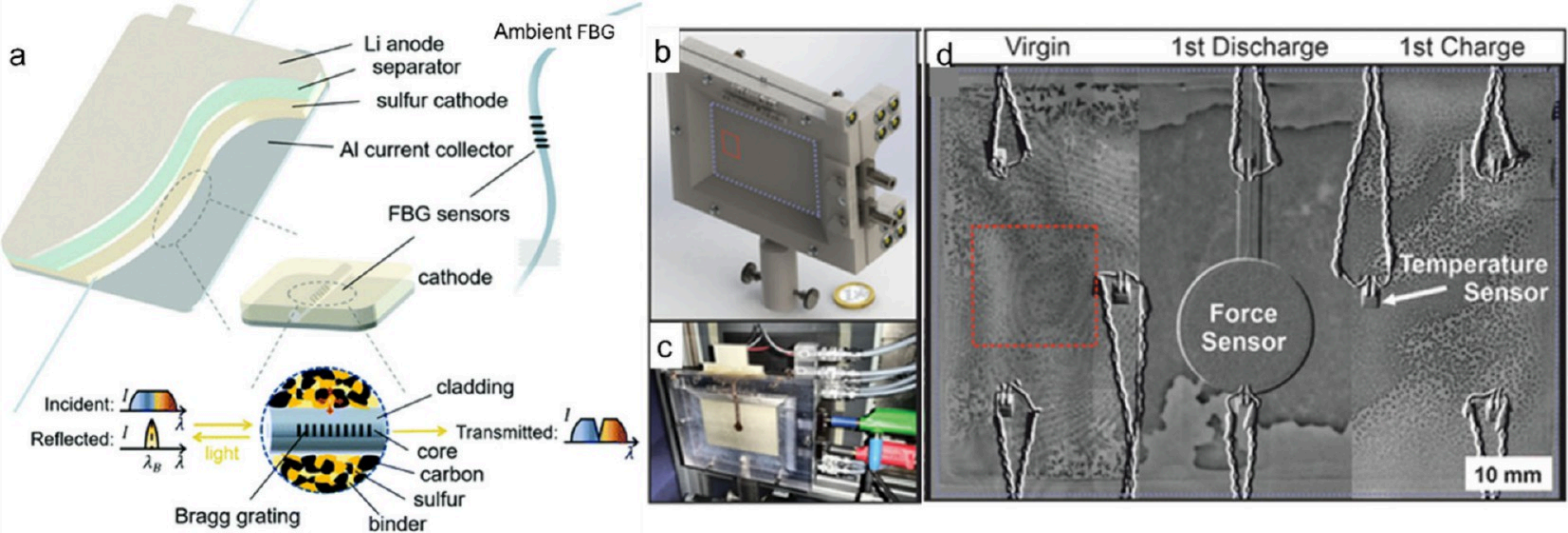
Recent sensor examples for monitoring gas, pressure, and temperature evolutions. (a) Schematic illustration of the structure of an FBG and a Li–S pouch cell embedded with an FBG. Reproduced with permission from ref (196). Copyright 2022, The Royal Society of Chemistry. (b) 3D image of setup design of multimodal analysis for pouch cells, (c) assembled setup optical photo, and (d) three sections of radiography images: Initial state (left), discharged state (middle), and charged state (right) from the marked full cell image (blue-dashed rectangle in (b), focus view image region (red-dashed rectangle in the virgin region), force sensor (marked in the middle panel), and temperature sensors (marked as arrow in the right panel). Reproduced with permission from ref (31). Copyright 2022, Wiley-VCH.

Chinnam et al. investigated the failure mechanisms of Li–S pouch cells using *operando* pressure analysis, placing the pouch cell between two external plates and using load cells to measure the pressure evolution.^195^ Their study provided valuable insights into optimizing Li–S pouch cell design and identifying strategies to enhance cell capacity and cycling performance through the application and monitoring of pressure. They observed that the Li-metal anode predominantly influences the thickness variation of the entire pouch cell. Moreover, their findings demonstrated that, beyond stabilizing the anode, applying high pressure improves cathode connectivity and mitigates cathode cracking during cycling. This, in turn, enhances the potential for developing high-sulfur mass-loading cathodes. In a similar direction, Miao et al. *in situ* monitored the internal mechanical stress evolution in Li–S pouch cells, which results from the continuous volume expansion and contraction during multiphase electrochemical reactions, by embedding lightweight optical fiber sensors within sulfur-based cathodes (Figure 12a).^196^ These optical fibers, which are highly resistant to both electromagnetic interference and chemical corrosion, provide real-time insights into the dynamic mechanical behavior of the cathode, further contributing to the understanding and optimization of sulfur-based battery performance.

At the commercial level, the reliance on direct contact to measure mechanical displacement, while maintaining a small form factor and ensuring compatibility with the harsh in-cell environment, presents challenges for implementing reliable sensing techniques. This often requires significant cell modifications and the use of external attachments, which can make such methods impractical for real-world applications.^197,198^ One promising approach involves the use of fiber-Bragg-grating (FBG) technology,^199^ which has been successfully applied to reduce the need for extensive cell alterations. However, FBGs are inherently sensitive to multiple factors, such as temperature, strain, and pressure, simultaneously, necessitating sensitivity tuning and complex data deconvolution to extract accurate pressure data. Microelectro-mechanical systems (MEMS)^200^ present a more robust and scalable alternative. These systems integrate small mechanical and electronic components onto a single microchip, and they can be arranged or embedded into a flexible sensing array. This approach allows for the combination of discrete sensing capabilities, such as temperature, pressure, and strain, into a single multifunctional platform. However, a common challenge across all these techniques is the need for efficient data extraction from inside the battery. Current methods rely on conductive wires or fiber optics to transmit data, but advancements in wireless^201^ or powerline^202^ data transmission systems could eventually eliminate the need for such physical connections, offering a more practical solution for commercial battery systems.

### Temperature Monitoring

The battery’s temperature is regulated by the thermal balance between heat generation and dissipation. The energy lost during the charging and discharging processes, often associated with parasitic exothermic reactions, can locally and globally increase the sulfur-based battery temperature. A sudden heat rise can indicate battery malfunction, potentially leading to thermal runaway, a dangerous condition in which an overheated battery triggers runaway reactions in adjacent cells, creating a chain reaction that may result in system failure or hazardous conditions. In some cases, heat can also be applied intentionally from an external source to reach a critical temperature for specific purposes, such as self-healing of the battery.

Seo et al. developed isothermal microcalorimetry and accelerating rate calorimetry techniques to investigate the thermal behavior of three LSB cathode materials at varying discharge rates.^203^ They employed a continuum model to calculate both the reversible entropic heat and irreversible resistive heat generated during discharge. By comparing the model data with experimental results, they assessed the contributions of reversible and irreversible heat to the total heat generation. The study found that the battery with the S–LiV_3_O composite cathode had the highest thermal runaway onset temperature and the lowest maximum self-heating rate.

To achieve multidimensional monitoring of cathode evolution, Müller et al. developed a system for Li–S pouch cells capable of simultaneously measuring temperature distribution, stack pressure, EIS, and XTM during discharge (Figure 12b–d). This multimodal approach provides an internal view of material transformations during cycling. By combining these four independent measurements, XTM for electrode morphology changes, EIS for the solution and charge transfer resistance, temperature distribution for heat dissipation, and force measurement for mechanical “breathing”, the system thoroughly analyzes the evolution of LSB pouch cells during operation.^31^

At the commercial level, accurate thermal mapping of battery cells presents challenges due to factors like skin-core heat gradients, mechanical and chemical constraints,^204^ and the balance between cost, size, and measurement accuracy. Sulfur-based batteries, as complex multilayer devices, often suffer from poor monitoring resolution and accuracy. One approach to overcoming these issues is the use of low-profile temperature sensors, either embedded directly into the cells during manufacturing or retrofitted later, to form distributed thermal sensing arrays.^205,206^ These arrays enable the creation of thermal maps during battery operation. The small size and high accuracy of the SMD thermistors used allow for such strategic placement inside the battery without affecting its operation. Inkjet printing^204,207^ offers another alternative, enabling even lower-profile sensing with sub-25-μm thickness arrays, though with a larger footprint. Either solution can be adapted to various cell formats, enhancing thermal diagnostic capabilities in industrially relevant cell formats. FBG technology has also been explored by various research groups, offering high-accuracy distributed thermal data.^208−211^ Its small size and nonconductive nature make it suitable for in-cell implementation. However, challenges remain with mechanical fragility and multiphenomena sensitivity, requiring special handling, tuning, and complex data deconvolution to ensure accurate monitoring.

To predict impending thermal runaway, a scalable solution involves detecting sudden changes in voltage, current, and temperature profiles. Fiber optic sensor technology can also provide early warnings of imminent thermal runaway by detecting these subtle shifts before a catastrophic failure occurs. This enables safety measures such as issuing warnings or initiating automatic shutdowns, allowing for intervention before the situation escalates to dangerous levels.

For any additional state tracking functions in battery cells, it is crucial that the sensors deployed are capable of enduring the challenging *in situ* environment and the rigorous manufacturing processes of batteries to ensure reliable, long-term monitoring. The sensors must be designed and processed to withstand the harsh conditions specific to sulfur-based battery cells, such as exposure to high temperatures, mechanical stress, and reactive chemicals. Embedding of sensors in or between the cell components, such as the current collector, separator, and enclosure, requires exploration of flexible yet robust substrates. To ensure chemical robustness, e.g., against electrolytes with polysulfides, sensors need to be conformally protected, either by coating or choosing chemically neutral materials, to minimize potential morphological, mechanical, and functional changes to the sensors as well as to the battery system itself.^212−214^

## Molecular Dynamic (MD) Simulations

Beyond the experimental findings obtained in the laboratory, a theoretical perspective is crucial for steering battery design strategies more effectively and unraveling the underlying mechanisms. MD simulations are a powerful computational method that models the movements of atoms and molecules over time and space by calculating intermolecular forces under various interactions. With advancements in computational capabilities and techniques, MD simulations have emerged as indispensable tools for enhancing the understanding and optimization of dynamic systems across a range of fields.^215^ In the field of sulfur-based batteries, MD simulations have proven effective in studying cathode deformation, lithium sulfide lithiation, ion diffusion coefficients, and lithium dendrite formation.^216^

These simulations allow researchers to explore micromechanisms that are difficult to observe directly in experiments, offering new theoretical foundations for battery design. For instance, the hybridization of sulfur with carbon-based nanomaterials has been shown to effectively suppress polysulfide dissolution.^217^ However, understanding how these nanomaterials influence sulfur utilization efficiency can be challenging with experimental methods alone. MD simulations provide crucial scientific insights, revealing the immobility of polysulfides,^218^ mitigating sulfur volume expansion,^219^ and enhancing battery capacity.^220^ Furthermore, the phenomena of polysulfide dissolution and the shuttle effect often occur within similar timeframes, complicating independent experimental investigations. MD simulations can elucidate the underlying micromechanisms of these complex intertwined processes, providing a deeper understanding and guiding more effective battery design strategies.^220^

MD simulations can analyze the adsorption and dissolution behavior of polysulfides on various cathode materials. By simulating these interactions, researchers can calculate the adsorption energies of polysulfides on nanocarbon materials or metal oxides, aiding in the optimization of cathode design. Additionally, MD simulations can utilize radial distribution functions (RDF) to investigate the aggregation and diffusion of polysulfides in the electrolyte, revealing their migration pathways and potential performance implications. Regarding the volume expansion of sulfur, MD simulations can provide insights into the volumetric changes of cathode materials at the nanoscale. Studies have shown that graphene and boron nitride nanowires can effectively mitigate sulfur expansion, thereby reducing capacity fade in the battery.

In LSBs, the electrolyte not only facilitates lithium ion conduction but is also the medium of polysulfide dissolution and migration. MD simulations play a significant role in investigating the ionic conductivity, polysulfide loss, and interfacial stability of the electrolyte. For instance, MD simulations can calculate the lithium-ion diffusion coefficients and conductivity within the electrolyte. Simulations of various solvent systems, such as DME and DOL, indicate that solvents with lower viscosity generally exhibit higher ionic conductivity, consistent with experimental results. The diffusion of polysulfides is a primary cause of capacity decay in LSBs. By utilizing DFT and MD simulations, researchers can compute the adsorption energies between polysulfides and electrolyte molecules, allowing for the optimization of electrolyte formulations to minimize polysulfide diffusion.

While MD simulations offer valuable insights into the research of sulfur-based batteries, they also face important challenges. First, high computational costs limit the simulation of large-scale systems, making it difficult for researchers to capture the complexities of battery environments. Second, MD simulations are typically constrained to time scales ranging from nanoseconds to microseconds, complicating the observation of long-term behaviors such as cycling stability and battery aging. Additionally, the sensitivity of simulation results to force field and model selection can impact the reliability of findings, leading to inconsistencies across different simulations. Future studies may integrate multiscale simulation methods to achieve a more comprehensive understanding of phenomena spanning from atomic to macroscopic scales. Moreover, the incorporation of machine learning techniques will significantly accelerate material discovery and performance prediction, allowing researchers to sift through vast data sets to identify optimization strategies. Through adaptive simulation methods, MD research can also dynamically adjust parameters to adapt to the evolving battery environment in real-time.

## Artificial Intelligence (AI)-Assisted Characterization

AI and ML have rapidly advanced over the past few decades, becoming integral in various scientific and industrial fields. These technologies are increasingly recognized as a cornerstone in material science research and all kinds of microscopies for instance.^221,222^ AI refers to the simulation of human intelligence in machines that are programmed to think and learn like humans. ML, a subset of AI, involves the use of algorithms and statistical models that enable computers to perform specific tasks without using explicit instructions, relying on patterns and inference instead.^223^

The integration of AI and ML in *in situ*/*operando* studies of batteries is becoming increasingly important due to the complexity and scale of data generated during these experiments. Acquiring, processing, and analyzing dynamic processes and chemical transformations within *operando* battery experiments can be challenging due to the massive amounts of data generated. Compared to traditional methods, AI offers several benefits, such as data analysis automation, which allows for fast processing of large volumes of data; transforming qualitative analysis into quantitative analysis, providing deeper insights into the behavior and performance of batteries; performing dimensionality reduction to accelerate data management and processing; and *in situ* experiments tracking, among other advantages. In the following paragraphs, we will discuss different types of AI/ML algorithms used for characterizing S-based batteries and *in situ*/*operando* experiments, which could represent future pathways for *in situ*/*operando* studies of S-based batteries.

### Mapping, Clustering, and Classification of Cathode Species

ML offers significant advantages in data treatment. Particularly, in *in situ*/*operando* studies, the automation of routines to obtain quantitative results is crucial due to the large volume of data generated by these techniques. In the case of sulfur-based batteries, a notable challenge is the automatic detection and classification of various species formed in the cathode during the charge and discharge processes. For the quantitative crystal phase mapping of Li vs Li_2_S, unsupervised algorithms such as PCA can be used in XRD, EELS, and 4D-STEM spectral data. PCA is a data decomposition algorithm that simplifies data into a few uncorrelated variables. By carefully selecting the number of variables, it is possible to map the relative composition of each phase in both time and space. PCA has also been used to map polysulfide intermediates in XAS data during a full discharge/charge cycle in LSBs and Ca–S batteries.^164,224^ However, the physical interpretation of this technique can be challenging because the components can present unphysical negative counts in the resulting spectral components. A comparable algorithm that overcomes this issue is non-negative matrix factorization (NMF), which ensures all components are positive throughout the spectral range, facilitating correlation with physical data.

For classifying polysulfide intermediates, PCA and NMF algorithms can be used in Raman and XAS data to decompose the information into components. Subsequently, clustering algorithms like K-means, fuzzy c-means, or K-nearest neighbors can be applied in these reduced spaces to classify the formation of different sulfur intermediates during the charge–discharge processes.^225,226^ While these unsupervised approaches are useful, they sometimes have limitations in accuracy and generalization. To overcome these limitations, supervised algorithms can be used. Their main drawback is their requirement of experimental labeled data for model training. However, in some experimental techniques, this is not a problem because such data is available. For instance, Cuisinier et al. obtained reference standards of S_8_, S_6_^2–^, S_4_^2–^, S_3_^2–^, and S^2–^ spectra for *in situ* XAS experiments.^112^ Similarly, Xu et al. prepared UV–vis data references for the five polysulfide species (Li_2_S_2_, Li_2_S_3_, Li_2_S_4_, Li_2_S_6_, and Li_2_S_8_).^227^ Using these references, a support vector machine algorithm can classify the intermediate species during *in situ*/*operando* experiments. Support vector machine works by finding the hyperplane that best separates different classes in the feature space.^228^

In the absence of experimental data, an alternative, though not as optimal, is to use simulated data for training these models. Aguiar et al. employed data from over 500,000 simulated crystals from different databases to train a CNN for phase classification in diffraction-TEM data.^229^ Nevertheless, the drawback of using simulated data for training is that the model may not capture all the variability and nuances present in experimental data. In contrast, it allows for the generation of much larger data sets for training the neural networks when the path toward experimental data generation is prohibitively resource-consuming.

### Automated Segmentation of S-Based Nanoparticles

One of the most interesting applications of ML in S-based batteries is the study of the size and morphology of the sulfur-based domains created during the discharge–charge process. Automated segmentation algorithms are key to obtaining quantitative results from *in situ/operando* micrographs. In this context, computer vision algorithms, like edge detection, and clustering algorithms, have been used for segmentation routines in STEM and SEM images.^230^ However, supervised algorithms, particularly those based on deep learning (DL), have proven to be the most accurate and effective. The segmentations provide 3D information, such as morphology and orientation, of the nanoparticles. Another widely used model in the field of microscopy that deserves special mention is the U-Net model, a CNN-based model with an encoder-decoder architecture. Several studies have shown this model to yield promising results in AFM, SEM, and STEM data.^231−233^ Parallel to these studies, R. Larsen et al. developed the Nanoparticles SAM (NP-SAM) model, a specialized adaptation of Meta’s (Facebook) state-of-the-art Segment Anything Model (SAM). This refined version is tailored for the detection and segmentation of nanoparticles in HAADF and BF-STEM images. This model surpasses the accuracy of more traditional approaches and improves the handling of overlapping particles, one of the most challenging aspects of nanoparticle segmentation and a common struggle for other models.^234^

### Process Optimization and Dimensionality Reduction

All the algorithms presented before had the same global objective: the automation of *in situ*/*operando* data treatment. By automating these processes, these algorithms provide scientists with powerful tools to extract quantitative results from large data sets in a significantly reduced analysis time. The next step in this data treatment optimization is to accelerate the computation of these processing algorithms. To achieve that, two different approaches can be followed. The first one is to optimize the efficiency of traditional algorithms using ML. For instance, one of the most time-consuming processes in this field is 3D reconstruction in tomography data. Moreover, *in situ* adds the time dimension to the reconstruction. Therefore, reconstructing in both space and time can be extremely slow without using high-performance computing, requiring substantial resources for a single sample.^42,235^ To optimize this process, Bladt et al. used an NN in electron tomography (ET) data, demonstrating that with only 10 projections, the NN model achieved results similar to the simultaneous iterative reconstruction technique algorithm, a traditional method in tomography, but with 151 projections. This significantly accelerates calculations by reducing the data used for final processing.^236^

The second approach, and the most direct one, is to reduce redundancy in data, typically achieved through dimensionality reduction using PCA or NMF. In spectroscopy techniques such as EELS, XRD, Raman, and XAS, PCA reduction is widely used as a preprocessing step.^237−240^ This process involves extracting the most relevant components and projecting the data into the resulting variable space, which not only considerably reduces the dimensions but also the noise, enhancing in this way the quantitative accuracy of subsequent processing. Going a step further, autoencoders (AEs) are emerging as a powerful dimensionality reduction model. AEs are NN-based models that encode image or spectral data into a small latent space that can later be used to reconstruct the original data. This latent space can encapsulate the physical properties of the data, enabling operations within this lower-dimensional representation and significantly accelerating calculations.^221^

### Automation and Acceleration of Data Acquisitions

Shifting focus from data treatment to data acquisition, there are also numerous opportunities to apply ML techniques in this area. One of the most prominent applications is the automation and acceleration of data acquisition processes. For certain *in situ* techniques mentioned earlier, some level of automation is already in place, allowing systems to operate independently once the experimental conditions are established. However, techniques like *in situ/operando* STEM, *in situ* AFM, and *in situ*-ET still require operator input during the experiment, such as exploring regions of interest and fine-tuning microscope conditions over time.

Such techniques can be used not only for automation but also for accelerating the acquisition process. One potential application is in *in situ*/*operando* tomography of sulfur batteries. Collecting tomographic series during *in situ/operando* experiments can be time-consuming, especially when aiming to capture all transition phases during charge/discharge cycles, which often results in large volumes of redundant data. By employing these models to detect significant changes in the material, one could automate the acquisition of tomographic series, thereby reducing both the generated volume of data and acquisition times. Furthermore, using CNN models for tomography reconstructions with fewer projections, as described in the previous section, can indirectly accelerate the acquisition process. In other words, this approach can reduce the number of tomographic scans needed to achieve high-quality 3D reconstructions.^236^

Overall, while the application of AI and ML in the study of sulfur-based batteries is still in its early stages, the advancements made in other materials and *in situ/operando* experiments offer valuable insights and highlight potential benefits for this field. Although AI and ML techniques have not yet been extensively applied to sulfur-based batteries, the methodologies currently in use have already demonstrated significant advantages, such as automating and accelerating data processing, enhancing accuracy, and optimizing experimental workflows. By leveraging these established techniques and adapting them to the unique challenges of S-batteries, it is possible to achieve more efficient and quantitative results. Therefore, integrating AI and ML into *in situ*/*operando* studies of sulfur-based batteries is not only a promising pathway but an essential one for advancing our understanding and improving the performance of these energy storage systems. The continued exploration and application of AI and ML will be crucial for overcoming existing limitations, unlocking new capabilities, and driving innovation in the study of sulfur-based battery technologies.

## Conclusion and Outlook

In conclusion, this work presents a comprehensive review of the advanced characterization techniques used in *in situ/operando* analysis of sulfur-based batteries, highlighting critical methods for detecting morphology, phase transitions, chemistry, polysulfide composition, and interfacial dynamics. By integrating these experimental techniques with dynamic theoretical calculations and the rapidly advancing fields of AI and ML, a more holistic understanding of the complex electrochemical mechanisms governing sulfur-based batteries is achieved. This synthesis of experimental and computational methods sheds light on the intricate processes at play, paving the way for significant improvements in battery capacity, efficiency, stability, and sustainability. By unraveling the challenges that have hindered the development of more efficient and durable sulfur-based energy storage systems, this approach positions these batteries as key candidates for next-generation energy storage technologies, advancing their potential for large-scale industrial production and broad application.

The *in situ/operando* characterization of sulfur-based batteries presents several key challenges that must be addressed to unlock their full potential in energy storage applications. Overcoming these obstacles requires a concerted effort to enhance experimental techniques, data processing, and integration with computational tools. Future research should prioritize improving both spatial and temporal resolution while effectively combining multiple techniques, potentially applying them within a single cell, and when possible, simultaneously gaining a more comprehensive understanding of battery processes. Efforts must also advance toward developing cells and experimental setups and conditions that accurately reflect real-world operating conditions, such as those with high sulfur loadings. Beyond *in situ* analysis, *operando* characterization under real conditions will provide even more valuable insights into the performance and degradation mechanisms of lithium–sulfur batteries. Furthermore, it is crucial to assess and minimize the impact of probing methods on the cell materials and performance. Stronger integration of theoretical models with experimental measurements is also essential. AI will undoubtedly play a pivotal role in the future of *in situ/operando* experiments, and it could also contribute to enhancing the efficiency of theoretical calculations. Below are the main challenges and areas for future development:

### Improving Resolution

One significant challenge is enhancing the spatial and temporal resolution of *in situ/operando* techniques. The complex nature of sulfur-based batteries, with their dynamic reactions, phase changes, and intricate interfacial processes, requires high-resolution and at the same time fast methods to capture fine details. Enhancing the resolution of current imaging and spectroscopy techniques will enable researchers to visualize and quantify the behavior of sulfur species and degradation mechanisms with unprecedented precision and detail.

### Combination of Techniques

No single technique can fully capture the complex, multifaceted nature of sulfur-based battery operation. The challenge lies in integrating multiple complementary techniques to obtain a holistic view of both chemical and structural changes during battery cycling. While some progress has been made, three critical areas for improvement remain to achieve a comprehensive understanding of how different processes interact with one another in real-time: (i) developing experimental setups capable of simultaneously combining different measurement techniques during the same battery cycle; (ii) developing cells compatible with multiple techniques; (iii) enhancing the ability to synchronize data from different sources and time scales.

### Toward the Analysis of Real Samples in Real Conditions

Efforts must also advance toward the development of cells, experimental setups, and testing conditions that accurately replicate real-world operating environments, such as those involving high sulfur loadings and realistic cycling parameters. Ideally, measurements should be conducted on actual, fully assembled batteries or the closest possible models. Additionally, different battery formats, such as coin-type and pouch-type cells, present distinct challenges that merit evaluation to fully understand their behavior and limitations. This shift is essential for obtaining results that are more representative of actual battery performance and durability.

### Demanding Experimental Conditions for *Operando* Studies

Beyond in situ analyses, operando characterization, where the battery should be studied under real-world operating conditions, remains particularly challenging for some of the techniques. The proposed reaction mechanism of batteries entails multiple reactions and adsorbed species, with some being rate-limiting while others exhibit rapid kinetics. Thus, the state of battery chemistry is determined by parameters such as scan rate, constant current, or frequency applied through conventional electrochemical methods like CV, galvanostatic charge and discharge tests, or EIS. During operational conditions, the temporal and spatial resolution of the technique must be suitable for capturing the process of interest or the lifetime of the species we aim to observe under non steady state conditions. Aside from cells resembling real working devices and fast-enough characterization techniques, we need to ensure the proper activation of the material before cycling, taking into account the effect of test conditions, such as the long time explosion under X-rays, electrons, etc. Additionally, conversion-type sulfur-based batteries are complex systems that evolve with cycling at time scales going from hours to years, thus we need to ensure the representativity of the analyzed cycle. Moreover, it is critical to assess and minimize the impact of probing methods, such as electron and X-ray beams, on the cell materials and overall performance. Reducing these artifacts will ensure that the measurements reflect the true behavior of the battery, leading to more reliable insights and guiding more effective design improvements.

### Integration of Experimental Results with Theoretical Models

Integrating experimental data with results from theoretical calculations, such as molecular dynamics simulations, presents also a significant challenge due to the different scales often involved. Experimental techniques provide real-time insights into battery behavior under operational conditions, capturing macroscopic and mesoscopic changes in structure, chemistry, and performance. However, theoretical models, especially ab initio and molecular dynamics simulations, focus on atomic or molecular-level interactions, often requiring simplifications that may not fully capture the complexity of real-world systems Bridging this gap begins with a rational definition of the theoretical model to ensure its relevance to the experimental context. Moreover, developing multiscale models that connect atomistic and molecular details from simulations with the larger-scale phenomena observed in experiments is crucial. Synchronizing the differing timeframes of experimental data and simulation dynamics is another critical hurdle, as real-time battery operation occurs on scales that computational models struggle to replicate. Achieving seamless integration will require advancements in both computational methods and experimental protocols, along with the use of AI and ML to streamline the comparison and alignment of data from these two complementary approaches.

### Integration of Experiments and Theory with AI/ML Algorithms

The vast amounts of data generated by *in situ/operando* experiments can be overwhelming, requiring sophisticated processing techniques to extract meaningful insights. Developing advanced data analysis pipelines capable of efficiently handling large data sets while integrating with theoretical models and simulations is a major challenge. AI and ML algorithms hold great promise in automating both data processing and acquisition, allowing for improved resolutions, identification of hidden patterns, and the selective capture of only meaningful data. However, their full capabilities in these areas have yet to be fully realized. The real challenge lies in effectively incorporating them into experimental workflows, developing reliable methods to fuse real-time experimental data with predictive models, and enabling dynamic adjustments to experimental parameters based on ongoing analysis. Developing better tools for data processing, pattern recognition, and real-time feedback during experiments will be essential for maximizing the utility of these characterization techniques.

Overall, *in situ* and *operando* characterization techniques are essential for advancing high-performance sulfur-based batteries, providing critical insights into the factors that influence battery performance and degradation. By uncovering these key mechanisms, researchers can more effectively guide the design of novel materials and optimize cell configurations, paving the way for significant improvements in capacity, efficiency, and stability. Ultimately, these advanced experimental techniques, properly integrated with theoretical calculations and AI tools, will help unlock the full potential of sulfur-based batteries, accelerating their path to commercialization and broadening their applications in next-generation energy storage solutions.
